# Interpreting Randomized Controlled Trials

**DOI:** 10.3390/cancers15194674

**Published:** 2023-09-22

**Authors:** Pavlos Msaouel, Juhee Lee, Peter F. Thall

**Affiliations:** 1 Department of Genitourinary Medical Oncology, The University of Texas MD Anderson Cancer Center, Houston, TX 77030, USA; 2 Department of Translational Molecular Pathology, The University of Texas MD Anderson Cancer Center, Houston, TX 77030, USA; 3 David H. Koch Center for Applied Research of Genitourinary Cancers, The University of Texas MD Anderson Cancer Center, Houston, TX 77030, USA; 4 Department of Statistics, University of California Santa Cruz, Santa Cruz, CA 95064, USA; juheelee@soe.ucsc.edu; 5 Department of Biostatistics, The University of Texas MD Anderson Cancer Center, Houston, TX 77030, USA; rex@mdanderson.org

**Keywords:** blocking, hazard ratios, confidence intervals, generalizability, randomized controlled trials, random allocation, random sampling, random treatment assignment, stratification, transportability

## Abstract

**Simple Summary:**

We provide an extensive review of the fundamental principles of statistical science that are needed to accurately interpret randomized controlled trials (RCTs). We use these principles to explain how RCTs are motivated by the powerful but strange idea that flipping a coin to choose each patient’s treatment is the most statistically reliable way to compare treatments. Random treatment assignment ensures fair comparisons between treatments because it does away with bias and confounding from variables other than treatment. If the goal is to estimate treatment effects in a patient population, rather than compare two or more treatments, then random sampling, not random treatment assignment, is required. However, random sampling is virtually impossible to carry out in a clinical trial because patients are accrued over time as they arrive in the clinic, subject to a trial’s entry criteria. Consequently, in practice, a trial provides a nonrepresentative convenience sample. Valid treatment comparisons provided by RCT data subsequently require additional causal assumptions of transportability of between-treatment effects from the sample to the patient population of interest. This may be used as a basis for choosing treatments for future patients. The present paper discusses what this means for practicing physicians who encounter RCT data in the literature.

**Abstract:**

This article describes rationales and limitations for making inferences based on data from randomized controlled trials (RCTs). We argue that obtaining a representative random sample from a patient population is impossible for a clinical trial because patients are accrued sequentially over time and thus comprise a convenience sample, subject only to protocol entry criteria. Consequently, the trial’s sample is unlikely to represent a definable patient population. We use causal diagrams to illustrate the difference between random allocation of interventions within a clinical trial sample and true simple or stratified random sampling, as executed in surveys. We argue that group-specific statistics, such as a median survival time estimate for a treatment arm in an RCT, have limited meaning as estimates of larger patient population parameters. In contrast, random allocation between interventions facilitates comparative causal inferences about between-treatment effects, such as hazard ratios or differences between probabilities of response. Comparative inferences also require the assumption of transportability from a clinical trial’s convenience sample to a targeted patient population. We focus on the consequences and limitations of randomization procedures in order to clarify the distinctions between pairs of complementary concepts of fundamental importance to data science and RCT interpretation. These include internal and external validity, generalizability and transportability, uncertainty and variability, representativeness and inclusiveness, blocking and stratification, relevance and robustness, forward and reverse causal inference, intention to treat and per protocol analyses, and potential outcomes and counterfactuals.

## 1. Introduction

The goal of a randomized controlled trial (RCT) is to generate data that can be used to compare treatments fairly, which in turn may guide patient-centered medical decisions [1,2,3,4]. Worldwide, results of approximately 140 RCTs are published each day, comprising an immense compendium of data that can be daunting for clinical practitioners and other stakeholders to digest efficiently [5]. As RCT designs and their data structures become more complex, the medical research community may be best served by focusing on the most informative signals, while avoiding noisy statistics and invalid inferences. To help keep inferences principled and useful, the present article focuses on the most essential components of RCTs. Medical RCTs are experiments with human subjects that are designed primarily to yield inferences about comparative causal treatment effects. In this article, we first describe the fundamental principles of statistical science, which we then use to explain how the action of random allocation of interventions justifies some, but not all, inferences and probability calculations.

Modern statistical science has evolved as a collection of models, methods, and computational algorithms for designing experiments, obtaining representative samples, performing computer-based simulation studies, constructing graphical displays, and analyzing a wide array of different data structures to make inferences about parameters of interest. In medical research, this includes methods for assessing how clinical outcomes, such as survival time, may be associated with treatments and patient characteristics based on different types of studies, such as clinical surveys or interventional trials. These methods can be divided into those related to sampling theory or experimental design [6,7,8].

## 2. Sampling Theory and Experimental Design

Sampling methods specify how to obtain a statistical sample, which is a set of objects from a population that one wishes to learn about, that reliably represents the population [6,7]. Inferences about the population are based on sample statistics, which are computed from the sample’s data using well-defined formulas or algorithms. A sample mean, correlation, or effect of a covariate on an outcome variable is used to estimate corresponding population parameters, also known as estimands, which are conceptual objects that are almost never known. This requires a number of implicit or explicit assumptions, such as the appropriateness of the statistical models for the type of data being analyzed, and the absence of unknown biases, data recording errors, or selective analysis reporting [9,10,11,12]. For simplicity, we will assume throughout this review that these statistical assumptions are correct unless otherwise stated. 

Denote a population parameter by θ an observable random variable by Y, and let P(Y|θ) denote an assumed probability distribution describing how Y varies in the population. A representative sample **Y** = {Y_1_, …, Y_n_} may be used to compute a statistical estimator of θ, and P(Y|θ) may be used to determine the estimator’s probability distribution. For example, a sample mean may be used to estimate a population mean. The distribution of the sample mean may be approximated by a normal distribution (bell-shaped curve) if the sample size is sufficiently large, and a 95% confidence interval (CI) around the observed sample mean may be computed to quantify uncertainty by giving us an idea of how closely we can estimate θ from the sample. Another example is that, given (X,Y) data on a numerical outcome variable Y and a covariate X, a regression model P(Y|X, α, β) with linear conditional mean E(Y|X) = α + βX may be assumed to characterize how Y varies with X. If Y is a binary (0/1) indicator of response, then a logistic regression model log{Pr(Y = 1|X)/Pr(Y = 0|X)} = α + βX can be used. In each case, the parameters θ = (α, β) may be estimated from a sample of (X,Y) pairs to make inferences about the population from which the sample was taken, provided that the sample accurately represents the population. For example, a *simple random sample* of size n must be obtained in such a way that all possible sets of n objects from the population are equally likely to be the sample. 

In contrast with sampling theory, experimental design involves statistical methods to plan a set of procedures, such as an RCT, to compare the effect of interventions, such as treatments, on outcomes of interest. Experimental design was largely pioneered by the English statistician, biologist, and geneticist Sir Ronald Fisher, who also invented RCTs, initially to maximize crop yield in agricultural experiments in the 1920s and 1930s. RCTs were popularized in medical research by the English epidemiologist and statistician Austin Bradford Hill in the 1940s and 1950s [13,14,15,16]. We will argue that, under appropriate assumptions, if one’s goal is to compare treatments as a basis for medical decision-making, then data from studies based on experimental designs that include randomization can be very useful.

## 3. Bayesian and Frequentist Inference

**Figure 1:**
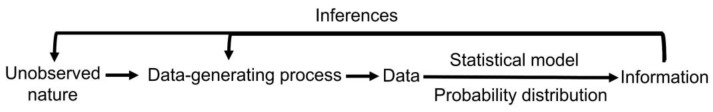
Information processing model of the two major schools of statistical inference. The unobserved collection of mechanisms in nature generates phenomena known as data-generating processes. These physical mechanisms generate data, which are then processed by statistical models that use probability distributions to generate information that can be quantified in binary digits (bits) of surprisal. Information can be used to make inferences about both the data-generating process and the unobserved underlying nature.

The results of medical studies can be analyzed using either frequentist or Bayesian statistical methods (Figure 1) [17]. These are two different statistical philosophies for constructing a probability model for observable variables and parameters and making inferences. A frequentist considers parameters θ to be unknown and fixed. A Bayesian considers parameters to be unknown and random and therefore specifies a *prior probability distribution* P(θ) to describe one’s degree of belief or uncertainty about θ before observing data. Specifying a prior distribution based on pre-existing contextual knowledge may be a nontrivial task [18]. Prior distributions can be classified as either “noninformative” or “informative” [19,20,21]. Noninformative priors are also known variously as “objective”, “flat”, “weak”, “default”, or “reference” priors, and they yield posterior estimators that may be close to frequentist estimators. For example, credible intervals (CrIs) under a Bayesian model may be numerically similar to CIs under a frequentist model, although the interpretation of CrIs is different from that of CIs. Informative priors, sometimes known as “subjective” priors, take advantage of historical data or the investigator’s subject matter knowledge. “Weakly informative” priors encode information on a general class of problems without taking full advantage of contextual subject matter knowledge [20,21]. Bayesian analysis is performed by combining the prior information concerning θ [i.e., P(θ)] and the sample information {Y_1_, …, Yn} into the posterior distribution P(θ|Y_1_, …, Yn) using Bayes’ theorem. The posterior distribution reflects our updated knowledge about θ owing to the information contained in the sample {Y_1_, …, Yn}, and quantifies our final beliefs about θ. Bayesian inferences thus are based on the posterior. For example, if L is the 2.5th percentile and U is the 97.5th percentile of the posterior, then [L, U] is a *95% posterior CrI* for θ i.e., θ is in the interval [L, U] with a probability of 0.95 based on the posterior, written as Pr(L < θ < U|data) = 0.95.

**Figure 2:**
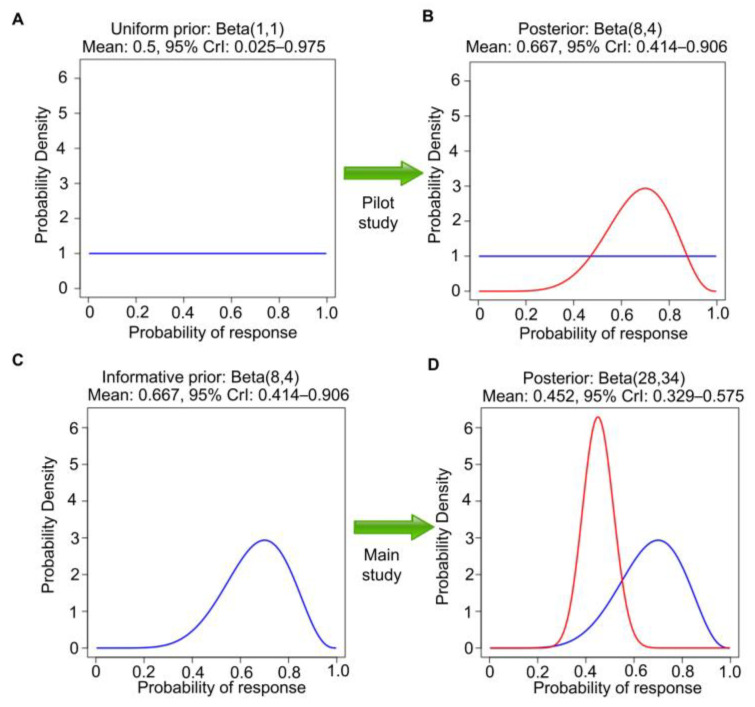
Bayesian updating of response probability to an investigational therapy in patients with chemotherapy-refractory renal medullary carcinoma (RMC). Prior probability distributions are colored blue and posterior probability distributions are colored red. (**A**) Uniform prior, also known as the Laplace prior, encoding the assumption that all response values in the unit interval of (0, 1) are equally likely. (**B**) Posterior probability distribution updated from the uniform prior after 7 out of 10 patients with RMC treated in a pilot feasibility study showed response. (**C**) Prior probability distribution encoding the knowledge obtained from the pilot study before conducting the main study. (**D**) Posterior probability distribution updated after 20 out of 50 patients with RMC treated in the main study showed response.

As a medical example of Bayesian inference, suppose that one is interested in the response probability that a new investigational therapy produces in chemotherapy-refractory renal medullary carcinoma (RMC). RMC is a rare, highly aggressive, molecularly homogeneous kidney cancer that lacks any effective treatment options [22,23,24,25]. To calculate a posterior distribution for Bayesian inferences, we can use the web application “Bayesian Update for a Beta-Binomial Distribution” (https://biostatistics.mdanderson.org/shinyapps/BU1BB/, accessed on 18 September 2023). This Bayesian model is useful for data consisting of a random number of responses, R, out of n independently sampled subjects, with the focus on θ = Pr(response), 0 < θ < 1. Let Y_1_, …, Y_n_ denote n patients’ binary response indicators, with Y_i_ = 1 if a response is observed from the ith patient and Y_i_ = 0 otherwise. We then have R = Y_1_ + … + Y_n_. Assuming conditional independence of n observations given θ, R follows a binomial distribution with parameters n and θ. A beta(a,b) distribution over the unit interval (0, 1) is a very tractable prior for θ. The beta(a,b) prior has mean a/(a + b) and effective sample size (ESS) = a + b, which quantifies the informativeness of the prior. The beta prior is commonly used because it is *conjugate* for the binomial likelihood; the posterior of θ given observed R and n is also a beta distribution, but with updated parameters, beta(a + R, b + n − R). In the RMC example, we define response as complete response (CR) or partial response (PR) on imaging at 3 months and assume beta(1,1) prior distribution, also known as Laplace’s prior. Beta(1,1) is the uniform distribution over the interval of (0, 1). That is, under beta(1,1), all values in the unit interval between 0 and 1 are equiprobable, and they can be viewed as noninformative (Figure 2A). It also has ESS = 2 and thus encodes little prior knowledge about θ. Because this cancer is rare and there is no comparator treatment, it is not feasible to conduct a randomized study. Suppose that a single-arm pilot study with n = 10 patients is conducted to establish feasibility and R = 7 responses are observed. This dataset allows us to update the uniform prior to the beta (1 + 7, 1 + 3) = beta(8,4) posterior, which has ESS = 12, posterior mean 8/12 = 0.67, and 95% posterior CrI 0.39–0.89 (Figure 2B). The Bayesian posterior estimator 0.67 *shrinks* the empirical estimate 7/10 = 0.70 toward the prior mean 0.50, which is characteristic of Bayesian estimation. Frequentist methods, such as those used in Least Absolute Shrinkage and Selection Operator (LASSO) or ridge regression, also achieve shrinkage by including penalty terms, a concept known as penalization [26]. In general, shrinkage and penalization improve the estimation of unknown parameters and enhance prediction accuracy. 

**Figure 3:**
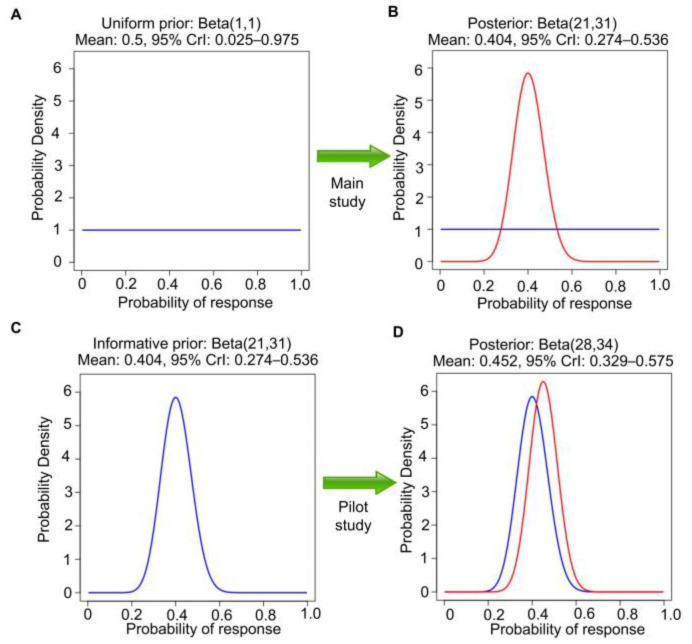
Bayesian updating of response probability to an investigational therapy in patients with chemotherapy-refractory renal medullary carcinoma (RMC). Prior probability distributions are colored blue and posterior probability distributions are colored red. (**A**) Uniform prior, also known as the Laplace prior, encoding the assumption that all response values in the unit interval of (0, 1) are equally likely. (**B**) Posterior probability distribution updated from the uniform prior after 20 out of 50 patients with RMC who were treated in the main study showed response. (**C**) Prior probability distribution encoding the knowledge obtained from the main study. (**D**) Posterior probability distribution updated after incorporating the results of the pilot study wherein 7 out of 10 patients with RMC showed response.

In general, Bayes’ Law may be applied repeatedly to a sequence of samples obtained over time, with the posterior at each stage used as the prior for the next. As a second step in the example, the beta(8,4) posterior can be used as the prior for analyzing a later single-arm study of this therapy in 50 new patients with chemotherapy-refractory RMC (Figure 2C). We assume that the second study also concerns the same θ = Pr(response). Suppose that 20 responses are observed in the second study. Then, the new beta(8,4) prior is further updated to a beta(8 + 20, 4 + 30) = beta(28,34) posterior. This has a mean 28/(28 + 34) = 0.45, with a narrower 95% CrI 0.33–0.58, reflecting the much larger ESS = 62 (Figure 2D). Let patient response indicators be denoted by Y_1_, …, Y_10_ for the pilot study and Y_11_, …, Y_60_ for the second study. Assume also that the subjects of the second study are sampled randomly from the same population as those of the first pilot study, a strong assumption that will be further explored in later sections. Furthermore, assume that Y_1_, …, Y_60_ are conditionally independent given θ. These assumptions allow the two Bayesian posterior computations described above to be executed in one step by treating Y_1_, …, Y_10_, Y_11_, …, Y_60_ as a single sample, assuming the first beta(1,1) prior, and directly obtaining the beta(28,34) posterior for θ in one step. If, instead, the second study were executed without observing the pilot study results, then it would be appropriate to use a uniform beta(1,1) prior, so 20 responses in 50 patients would lead to beta(1 + 20, 1 + 30) = beta(21,31) posterior. This has mean 21/(21 + 31) = 0.40 and 95% CrI 0.27–0.54 (Figure 3A,B). This is different from the posterior in Figure 2D because the two analyses begin with different priors, a beta(1,1) prior to seeing the pilot study results versus a beta(8,4) prior using the observed pilot study data. However, if the data from the pilot study are revealed afterward (Figure 3C,D), then the final posterior distribution will be the same as in Figure 2D. This is an example of the general fact that, if data are generated from the same distribution over time, then repeated application of Bayes’ Law is *coherent* in that it gives the same posterior that would be obtained if the sequence of datasets were observed in one study. 

## 4. Confirmations and Refutations

**Figure 4:**
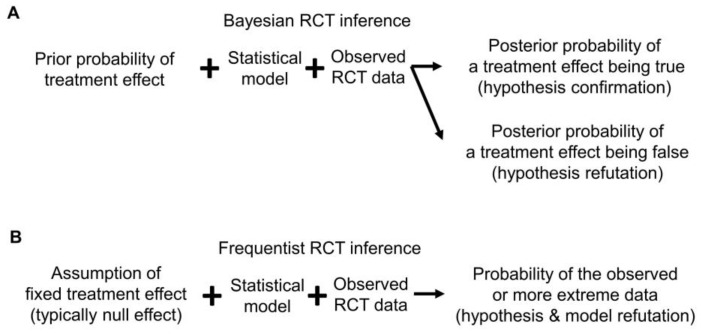
Frequentist and Bayesian Inference. (**A**) In a randomized controlled trial (RCT) testing a new therapy versus control, the null hypothesis is expressed as θ = 0 for the relative treatment effect difference between the new therapy and the control. Bayesian models can be used to obtain posterior probabilities of a treatment effect being correct relative to alternative treatment effect values (confirmationist inference) or wrong (refutationist inference). (**B**) Frequentist models do not use prior distribution but can be used to investigate purely refutational RCT evidence against the embedded statistical model and the assumption that the test hypothesis (typically the null hypothesis of no treatment difference) is true. For example, if the null hypothesis and all other model assumptions are true, the physical act of random treatment assignment would be expected to generate a random distribution of the data D yielded by repeated replications of the RCT. The amount of divergence of the observed data from this expected random distribution is a measure of refutational evidence against the null hypothesis that θ = 0 and all other underlying model assumptions. Similar considerations can be applied to generate refutational evidence against other tested hypotheses corresponding to different values of θ.

Bayesian posterior estimates may be used as evidence to either confirm or refute a prior belief or hypothesis. The former may be called “confirmationist” reasoning, which evaluates the evidence supporting a belief or hypothesis regarding specific values of a parameter (Figure 4A) [27,28]. For example, say that analysis of an RCT using the Bayesian survival regression model previously described in [29,30,31] yields posterior mean HR = 0.71 with 95% CrI 0.57–0.87 for overall survival (OS) time comparing a new treatment E to a control C. This may be interpreted as strong confirmational statistical evidence supporting the prior assertion that E is superior to C, formally that HR < 1. In contrast, “refutational” logic seeks evidence against a belief or hypothesis regarding a parameter value [32,33,34]. Using refutational logic, if the hypothesis is that E is inferior to C, formally that HR > 1, then a very small posterior probability Pr(HR > 1|data) can be interpreted as strong evidence against the belief that E is inferior to C (Figure 4A). Because Bayesian reasoning is probabilistic, it is different from logical conclusive verifications or refutations, such as exculpatory evidence that a suspect of a crime has an alibi, implying that it is certain the suspect could not have committed the crime [27,28]. The philosopher of science Karl Popper highlighted the asymmetry between confirmationist and refutational reasoning because evidence can only support (confirm) a theory in relation to other competing theories, whereas evidence can refute a theory even if we lack a readily available alternative explanation [33,34]. Therefore, refutational approaches require fewer assumptions than confirmationist ones.

Since frequentists assume that a parameter is fixed and unknown, for example in a test of the frequentist hypothesis H_0_: HR = 1.0 of no treatment difference, no probability distribution is assumed for the parameter HR. A frequentist test compares the observed value T^obs^ of a test statistic T to the distribution of T that would result from an infinite number of repetitions of the experiment that generates the data, assuming that H_0_ is true. If T^obs^ are very unlikely to be observed based on the distribution of T under H_0_, this serves as refutational evidence against H_0_ (Figure 4B). This can be quantified by a *p*-value, which is defined as 2 × Pr(T > |T^obs^) for a two-sided test, under specific model assumptions [35,36,37]. 

While *p*-values are used as refutational evidence against a null hypothesis, they are often misunderstood by researchers [35,38]. A pervasive problem is that “statistical significance” is not the same thing as practical significance, which depends on the context of the study. Furthermore, the arbitrary *p*-value cutoff of 0.05 is often used to dichotomize evidence as “significant” or “non-significant”. This is a very crude way to describe the strength of evidence for refuting H_0_ [39]. A practical solution for this problem is to quantify the level of surprise provided by a *p*-value as refutational evidence against a given hypothesis in terms of bits of information, which are easy to interpret. This can be executed by transforming a *p*-value into an S-value [12,40], defined as S = −log_2_(*p*). Bearing in mind that a *p*-value is a statistic because it is computed from data, if H_0_ is true then a *p*-value is uniformly distributed between 0 and 1. This implies that under H_0_, a *p*-value has a mean 1/2 and, for example, the probability that *p* < 0.05 is 0.05. The rationale for computing S is that the probability of observing all tails in S flips of a fair coin equals (1/2)^S^, so *p* = (1/2)^S^ gives S as a simple, intuitive way to quantify how surprising a *p*-value should be [12,41,42,43,44]. S represents the number of coin flips, typically rounded to the nearest integer. Suppose that an HR of 0.71 is observed and a *p*-value of 0.0016 is obtained against the null hypothesis of HR = 1.0. Since −log_2_(0.0016) = 9.3, rounding this to the nearest integer gives S = 9 bits of refutational information against the null hypothesis of HR = 1. This may be interpreted as the degree of surprise that we would have after observing all tails in 9 consecutive flips of a coin that we believe is fair. A larger S indicates greater surprise, which is stronger evidence to refute the belief that the coin is fair, which corresponds to the belief that H_0_ is true. Thus, the surprise provided by an S-value is refutational for H_0_. In this case, S = 9 quantifies the degree of surprise that should result from observing a *p*-value of 0.0016 if H_0_ is true. We provide a simple calculator (Supplementary File S1) that can be used by clinicians to convert *p*-values to S-values. Of note, S-values quantify refutational information against the fairness of the coin tosses, which includes but is not limited to the possibility that the coin itself is biased towards tails. An unbiased coin can be tossed unfairly to result in all tails in S flips. For simplicity herein, our assertion that the coin is “fair” encompasses all these scenarios.

Since S is rounded to the nearest integer, *p*-values 0.048, 0.052, and 0.06 all supply approximately 4 bits of refutational information, equivalent to obtaining 4 tails in 4 tosses of a presumed fair coin. A *p*-value of 0.25 supplies only 2 bits of refutational information, half the amount of information yielded by *p* = 0.06. While *p* = 0.05 is conventionally considered “significant” in medical research, it corresponds to only 4 bits of refutational information. This may explain, in part, why so many nominally significant medical research results are not borne out by subsequent studies. For comparison, in particle physics, a common requirement is 22 bits of refutational information (*p* ≤ 2.87 × 10^−7^), which corresponds to obtaining all tails in 22 tosses of a fair coin [45]. 

Converting *p*-values to bits of refutational information can also be very helpful for interpreting RCT results that have large *p*-values. For example, the phase 3 RCT CALGB 90202 in men with castration-sensitive prostate cancer and bone metastases reported an HR of 0.97 (95% CI 0–1.17, *p* = 0.39) for the primary endpoint, time to first skeletal-related event (SRE), using zoledronic acid versus placebo [46]. It is a common mistake, sometimes made even by trained statisticians [38], to infer that a large *p*-value confirms H_0_, which is wrong because a null hypothesis can almost never be confirmed. In the example, this misinterpretation would say that there was no meaningful difference between zoledronic acid versus control. The correct interpretation is that there was no strong evidence against the claim of no difference between zoledronic acid versus control in time to the first SRE. Using the S-value, *p* = 0.39 supplies approximately 1 bit of information against the null hypothesis of no difference, which is equivalent to asserting that a coin is fair after tossing it only once. This is why a very large *p*-value, by itself, provides very little information [35]. 

Conversion to bits of information can also help to interpret a frequentist 95% CI, which may seem counterintuitive due to its perplexing definition, which says that if the experiment generating the data were repeated infinitely many times, about 95% of the experiments would give an (L, U) CI pair containing the true value, assuming the statistical model assumptions are correct [47]. For example, an estimated HR of 0.71 with a 95% CI of 0.57–0.87 is obtained for HR in a hypothetical RCT study. A frequentist 95% CI, corresponding to a *p*-value threshold of 1 − 0.95 = 0.05, gives an interval of HR values for which there are no more than approximately 4 bits of refutational information, since S = −log_2_(0.05) ≈ 4, assuming that the statistical model assumptions are correct. Thus, the data from the RCT suggest that HR values within the interval bound 0.57 and 0.87 are at most as surprising as seeing 4 tails in 4 fair coin tosses. Values lying outside this range have more than 4 bits of refutational information against them, and the point estimate HR of 0.71 is the value with the least refutational information against it. Similarly, frequentist 99% CIs correspond to a *p*-value threshold of 1 − 0.99 = 0.01 and thus contain values against the null with at most −log_2_(0.01) ≈ 7 bits of information, which is the same or less surprising than seeing 7 tails in 7 tosses of a fair coin. A number of recent reviews provide additional guidance on converting statistical outputs into intuitive information measures [11,12,36,37,40,48].

Any statistical inferences depend on the probability model assumed for the analysis. This is important to keep in mind because an assumed model may be wrong. The Cox model assumes that the HR is constant over time, also known as the proportional hazards (PH) assumption. Unless otherwise stated, all the RCT examples we will use herein assume a standard PH model for their primary endpoint analyses. If the data-generating process is different from this assumption, for example, if the risk of death increases over time at different rates for two treatments being compared, then there is not one HR, but rather different HR values over time. For example, it might be the case that the empirical HR is close to 2.0 for the first six months of follow-up, but then is close to 0.50 thereafter. Consequently, inferences focusing on one HR parameter as a between-treatment effect can be very misleading, because that parameter does not exist. To avoid making this type of mistake, the adequacy of the fit of the assumed model to the data and the plausibility of the model for inferential purposes should be assessed. Whereas Bayesian inference focuses more on the coherent updating of beliefs based on observed data (Figure 2 and Figure 3), frequentist inference places more emphasis on *calibration*, i.e., that events assigned a given probability occur with that frequency in the long run. Furthermore, as reviewed in detail elsewhere [12,49], frequentist outputs, such as *p*-values, provide refutational evidence against all model assumptions, not only a hypothesis or parameter value (Figure 4B). Accordingly, frequentist outputs can be used directly to determine whether the distribution of the observed data is compatible with the distribution of the data under the assumed model. Thus, a small *p*-value implies that either H_0_ is false or that the assumed model does not fit the data well. For simplicity, hereafter we will follow the common convention used in medical RCTs of assuming that the model is adequate, and thus that a small *p*-value yields refutational evidence only against the tested hypothesis, which typically is the null hypothesis of no treatment difference. 

## 5. Inferences and Decisions

Although the term “evidence” does not have a single formal definition in the statistical literature, various information summaries are routinely used to quantify the strength of evidence [10,12,35,50]. These include frequentist parameter point estimates, CIs, and *p*-values. Estimation is the process of computing a statistic, such as a point estimate, interval estimate (such as frequentist CIs and Bayesian CrIs), or distributional estimates (such as Bayesian posterior distributions or frequentist confidence distributions), which aim to provide plausible values of the unknown parameter based on the data [12,17,51,52]. Statistical inference is a larger, more comprehensive process that involves using data not only to estimate parameters but also to make predictions and draw conclusions about a larger population based on a sample from that population (Figure 1). Causal inferences focus on estimating the effects of interventions [2,53]. An example of a frequentist causal inference can be obtained from the KEYNOTE-564 phase 3 RCT of adjuvant pembrolizumab versus placebo in clear cell renal cell carcinoma (ccRCC). The primary endpoint analyses for this RCT were based on the standard PH regression model often used in survival analyses of RCTs in oncology [1,2,54,55]. After a median follow-up of 24.1 months, the estimated HR for the primary endpoint, disease-free survival (DFS) time, was 0.68 with 95% CI 0.53–0.87 and *p* = 0.002 [54]. This corresponds to 9 bits of refutational information against the assumed model and null hypothesis that adjuvant pembrolizumab has the same mean DFS as placebo. Any HR values in the 95% CI 0.53–0.87 are less surprising than seeing 4 tails in 4 fair coin tosses, while values outside the CI have higher refutational information against them. 

The same data from KEYNOTE-564 can be analyzed to compare DFS times of adjuvant pembrolizumab versus placebo in ccRCC using a Bayesian framework. While noninformative priors that give numerical posterior estimates similar to frequentist estimates in the absence of multiple looks at the data [56] can be considered, informative priors may be used to incorporate prior information [21,57]. For example, a prior distribution may be formulated to account for the exaggeration effect, also known as the “winner’s curse”, often seen in reported phase 3 RCTs due to publication bias [29,30,31]. Phase 3 RCTs with negative results are less likely to be accepted for publication by the editors of medical journals [29], so the estimated effect sizes in published phase 3 RCTs are biased upward and thus are likely to overstate actual treatment differences [29,58]. This exaggeration effect due to the biased publication of studies with positive results is an example of a general phenomenon known as *regression toward the mean*, wherein, after observing an effect estimate X in a first study, upon replication of the experiment, the estimate Y from a second study is likely to be closer to the population mean [29]. Three recent studies [29,30,31] empirically analyzed the results of 23,551 medical RCTs available in the Cochrane Database of Systematic Reviews (CDSR), which provided an empirical basis for constructing an informative prior distribution that accounts for the anticipated exaggeration effect in published phase 3 RCTs [30]. If published pivotal phase 3 RCTs, such as KEYNOTE-564, are of sufficient quality to meet the criteria for inclusion in the CDSR, it is plausible to use the proposed prior, which may be called the “winner’s curse prior”, to account for the anticipated exaggeration effect [29,31]. 

**Figure 5:**
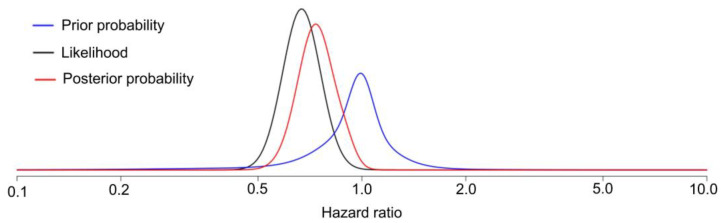
Bayesian updating of the DFS HR estimate of the KEYNOTE-564 phase 3 RCT that compared adjuvant pembrolizumab versus placebo in ccRCC. The informative prior probability distribution (blue) is designed to account for the winner’s curse based on an empirical analysis of the results of 23,551 medical RCTs of relative treatment efficacy available in the Cochrane Database of Systematic Reviews. The likelihood (black) is based on the reported frequentist results of KEYNOTE-564, demonstrating an HR of 0.68 with 95% frequentist confidence intervals of 0.53 to 0.87. The posterior distribution (red) combines the prior information (blue) and information from the data (black) and lies in-between. The resulting posterior distribution (red) accounts for the winner’s curse and yields a Bayesian posterior mean HR of 0.76 with 95% posterior CrI 0.59–0.96. The posterior probability that the HR is larger than 1.0 is 0.8%.

Recalling that the frequentist estimate of the HR for DFS time is 0.68 for the KEYNOTE-564 trial, the winner’s curse prior and model, described in detail elsewhere [29,30,31], gives a posterior mean HR of 0.76 with 95% CrI 0.59–0.96 (Figure 5), a substantial shrinkage of the frequentist estimate toward 1. This says that, under this prior and assumed statistical model, Pr(0.59 < HR < 0.96|data) = 0.95 (confirmationist inference), and Pr(HR > 1.0|data) = 0.008 (refutationist inference). A free web application is available (https://vanzwet.shinyapps.io/shrinkrct/, accessed on 18 September 2023) for clinicians to perform such Bayesian conversions of reported RCT data. 

Decisions may rely on statistical inferences, but they are not the same thing. A decision may be made by combining information from statistical inferences with subjective cost-benefit trade-offs to guide actions [10,59,60]. Such trade-offs may be expressed as type I and II error probabilities in frequentist tests of hypotheses, or by a utility function [1,59,60]. If an RCT were repeated infinitely many times, its type I error upper limit α quantifies the proportion of times that one would be willing to incorrectly reject a true null hypothesis of no difference between treatment and control. The type II error upper limit β is the proportion of times that one would incorrectly conclude that a false null hypothesis is correct when a particular alternative hypothesis is true. For example, in KEYNOTE-564, it was decided to set α = 0.025 for a test of the primary endpoint, DFS time. Because the estimated *p*-value was below this threshold, it was concluded that the result was “statistically significant” [54]. This approach is typically used to inform regulatory decisions by agencies such as the United States Food and Drug Administration (FDA) and the European Medicines Agency (EMA). Academic journals, by design, focus on publishing inferences, but they must make their decisions on which RCTs to publish based on various cost-benefit trade-offs that can include maintaining the journal’s reputation, as well as information on type I and type II error control [61]. Whereas estimations and corresponding inferences are quantitative and typically on a continuous scale, decisions usually are dichotomous, e.g., whether a statistical test is “significant” or “nonsignificant”, whether or not to approve a therapy, or whether to accept or reject an article in a journal. 

To illustrate the difference between inferences and decisions, we compare the results of the METEOR and COSMIC-313 phase 3 RCTs, which used a similar design and the same decision-theoretic trade-offs of type I error probability α = 0.05 and type II error probability β = 0.10 to guide tests of hypotheses for the primary endpoint of progression-free survival (PFS) time [62,63,64]. METEOR compared salvage therapies cabozantinib versus everolimus as a control in 375 patients with advanced ccRCC and reported an estimated HR of 0.58 (95% CI 0.45–0.75, *p* < 0.001) for the PFS endpoint [62]. COSMIC-313 compared the combination of cabozantinib + nivolumab + ipilimumab versus placebo + nivolumab + ipilimumab control as first-line therapies in 550 patients with advanced ccRCC. To date, this trial has an estimated HR of 0.73 (95% CI 0.57–0.94, *p* = 0.013) for the PFS endpoint [64]. Both results were declared “statistically significant” by the trial design because their *p*-values were lower than the conventional threshold of 0.05. They both supplied more than 4 bits of refutational information against the null hypothesis. However, METEOR yielded a far stronger PFS signal than COSMIC-313, since its reported *p*-value of <0.001 corresponds to at least 10 bits of refutational information against the null hypothesis, HR = 1, that cabozantinib has the same mean PFS outcome as everolimus. METEOR did not provide the exact *p*-value, but using established approaches [65], and the calculator provided in Supplementary File S1, we can back-compute the *p*-value using the reported 95% CIs to be approximately 3.5 × 10^−5^, which corresponds to 15 bits of refutational information against the null hypothesis. On the other hand, the *p*-value of 0.013 reported by COSMIC-313 supplied only 6 bits of refutational information against the null hypothesis that the triplet combination of cabozantinib + nivolumab + ipilimumab yields the same average PFS as the control arm. Therefore, although both trials were considered to show a “positive” PFS signal using the same *p*-value cutoff of 0.05 based on prespecified decision-theoretic criteria, METEOR yielded more than twice the refutational information against its null hypothesis compared with COSMIC-313.

Similar conclusions may be obtained if we examine the two trials using a Bayesian approach to generate posterior probabilities and CrIs. We assume that both METEOR and COSMIC-313 meet the criteria to be included in the CDSR and accordingly use the winner’s curse prior to reducing exaggeration effects [30]. For METEOR, the posterior mean HR = 0.65 with 95% CrI 0.49–0.83 and posterior probability Pr(HR > 1.0|data) = 0.00027, strongly favoring the cabozantinib arm over the everolimus control arm. Conversely, for COSMIC-313, the posterior mean HR = 0.81 with 95% CrI 0.63–1.01 and Pr(HR > 1.0|data) = 0.031 the control arm yielded better PFS than the triplet combination. Thus, when viewed through either a frequentist or Bayesian lens, the signal of METEOR is far stronger than that of COSMIC-313, despite both RCTs being reported as positive for their PFS endpoint. This illustrates the general fact that estimation yields far more information than a dichotomous “significant” versus “nonsignificant” conclusion from a test of hypotheses. Ultimately, decisions of which therapies to use in the clinic should incorporate each patient’s goals and values; account for trade-offs related to additional endpoints, such as OS, adverse events, quality of life, and financial and logistical costs; and account for individual patient characteristics [1].

## 6. Pre Hoc and Post Hoc Power

A concept related to decision-theoretic error control is the power, 1 − β, of an RCT. Because the type II error probability is a frequency-based computation for a selected specific value HR* under the alternative hypothesis (i.e., H_a_: HR = HR*), it is not used in the interpretation of a completed RCT. While there is typically only one null hypothesis, that the HR = 1.0, there are infinitely many potential alternative HR* values. Since a typical power computation is based on one arbitrary value for the alternative hypothesis and essentially is a device for computing sample size, most power computations have very little value and may be misleading after a trial has been completed. For example, the stated power of the CLEAR phase 3 RCT in metastatic ccRCC was determined based on the selected alternative value HR* of 0.714 for the primary endpoint of PFS, but upon completion of the trial, the estimated HR was 0.39 [66]. Post hoc power calculations conducted using the observed results after RCT completion are simply a re-expression of the observed *p*-value, and they provide no additional information [67]. This is the reason why knowing the power of an RCT is useful during the design stage of the trial, mainly as a rationale for sample size, but it has no value when analyzing the trial’s data. After the RCT is completed, the main interest for causal inferences is the uncertainty intervals of comparative parameters such as HRs or differences between means [35,67]. Due to the arbitrariness of HR*, it may be argued that a typical power computation is little more than a device to rationalize a computed sample size and that a plotted curve or table of power figures for a range of HR* values is much more honest and informative.

## 7. Variability and Uncertainty 

Statistical outputs may include descriptive summaries representing the *variability* of the data, such as the standard deviation (SD), range, or interquartile range (IQR) from the 25th to 75th sample percentiles. For example, among the patients treated with adjuvant pembrolizumab in KEYNOTE-564, the median age was 60 years with a range of 27–81. A sample range pertains to values observed in the patients enrolled in an RCT but is of limited use in making inferences about patient populations. Conversely, statistical summaries of *uncertainty*, such as a sample standard error (SE, also denoted by σ) or a CI, are used to make inferences about a parameter, such as the relative treatment efficacy of adjuvant pembrolizumab versus placebo. Variability is more general than *variance* [68], σ^2^, which is a parameter defined as the expected value of (Y *−* μ)^2^ for Y, a random variable of a population with mean μ. A population variance typically is estimated by a sample variance s^2^, which is often used to compute the SE = s/sqrt(n) of a sample mean. As the sample size increases, the SE decreases, and the CI for the mean becomes narrower [68,69,70]. 

## 8. Aleatory and Epistemic Probabilities

In frequentist inference, randomization ensures that uncertainty estimates of between-treatment effects will be unbiased. In Bayesian inference, randomization physically justifies the derivation of a randomization-based prior probability for the parameter of interest [71,72]. For example, the prior probability that a fair coin will land as heads is physically justifiable to be 0.5 [73]. Priors informed by known physical interventions, such as a fair coin flip, are called “aleatory” probabilities to distinguish them from “epistemic” probabilities, which are based on ignorance about the underlying data-generating process [73]. Aleatory and epistemic probabilities thus may coincide numerically, but they express very different concepts. For example, we may assign a prior probability of 0.5 for the outcome of a game of chess between two randomly chosen people [73]. This epistemic probability is not based on a well-defined underlying physical process but instead is derived from pure ignorance about the contestants [74]. The outcome of flipping a fair coin would be assigned a numerically equivalent prior probability of 0.5 but is based on a very well-understood data-generating process and thus would be expected to remain the same as we obtain more information. Conversely, epistemic probabilities can be updated as we gain more information. For example, our prior probability regarding who will win a game of chess will change if we find out that one of the contestants is a chess grandmaster. The distinction between epistemic and aleatory probabilities is subtle, but it stands at the heart of RCT inferences. Traditional frequentist RCT interpretations do not use epistemic probabilities and accept only aleatory probabilities as valid measures of uncertainty. Such aleatory probabilities are generated by physical procedures such as random sampling and random allocation. 

## 9. Random Sampling and Random Allocation

**Figure 6:**
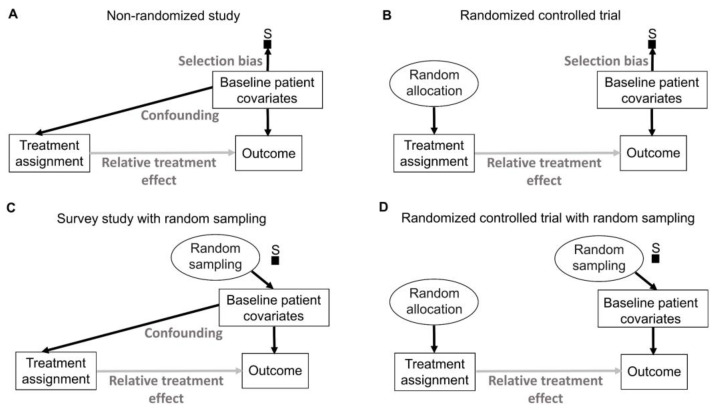
Selection diagrams distinguishing the causal effects of the two major types of random procedures used in research. (**A**) In a nonrandomized trial, the baseline covariates of patients can confound the estimation of the relative treatment effect because they can influence both treatment assignment and the outcome of interest. The selection node S indicates that sampling biases influence the enrichment of these baseline patient covariates in the study. (**B**) In an RCT, the treatment assignment of each patient or other study unit is only influenced by the random allocation procedure. Therefore, the baseline patient covariates can no longer be systematic confounders of the relative treatment effect but still influence the outcome, thus serving as prognostic factors. The physical act of randomization justifies the estimation of uncertainty measures as random errors for the relative treatment effect parameter comparing the enrolled groups (comparative inference). (**C**) In survey studies, the random sampling of patients from the population of interest removes systematic sampling biases and provide a physically justifiable distribution for the probability that the enrolled sample estimates for each sampled group are generalizable to the broader population. (**D**) In pure randomization inference, random allocation and random sampling remove systemic confounding and sampling bias thus allowing the physically justifiable estimation of uncertainty estimates for both the relative treatment effect and sample generalizability.

Random sampling and random allocation, also known as randomization, are both random procedures in which the experimenter introduces randomness to achieve a scientific goal. This is different from the randomness that an observable variable Y appears to have due to the uncertainty about what value it will take. The use of random procedures as an integral part of frequentist statistical inference to generate aleatory uncertainty estimates was pioneered by Ronald Fisher during the first half of the 20th century [75,76]. His insight can be represented explicitly by causal diagrams, as shown in Figure 6. We refer readers to comprehensive overviews for details on causal diagrams, which are used to represent assumptions about the processes that generate the observed data [2,77,78,79]. Figure 6 uses a type of causal-directed acyclic graph (DAG) known as a selection diagram, which includes a *selection node*, S, that represents selection bias when sampling from a patient population [2,80,81,82,83]. Selection DAGs can help to distinguish between the effects of random allocation (Figure 6B) and random sampling (Figure 6C). In RCTs, the focus is on the comparative causal effect, also known as the relative treatment effect, of a new treatment under investigation versus a standard control treatment. Each patient can only be assigned to one treatment, denoted by “Treatment assignment” in Figure 6. In nonrandomized studies, because physicians use patient covariates such as age, disease burden, or possibly biomarkers to choose a treatment assignment, this effect is denoted by the solid arrow from “Baseline patient covariates” to “Treatment assignment” in Figure 6A. The solid arrow from “Baseline patient covariates” to “Outcome” in Figure 6A denotes that these covariates may also influence the outcome and thus are *confounders* that can create a false estimated association between treatment assignment and outcome [2,77,78,79]. Random treatment allocation removes the causal arrow from any covariate, whether it is observed or not, to treatment and thus removes confounding (Figure 6B). Whether a study is randomized or observational, baseline patient covariates may directly influence the outcome, e.g., OS time, thus acting as prognostic factors. Because RCTs typically test the null hypothesis of no influence between treatment assignment and outcome, Figure 6 denotes this putative causal effect with a gray arrow. 

If a trial is designed to balance treatment assignments within subsets determined by known patient covariates, but it does not randomize, then other known or unknown covariates still may influence both the treatment assignment and the outcome, as shown in Figure 6A. Fisher’s insight was that all confounding effects can be removed and the uncertainty of the relative between-treatment effect can be estimated reliably by allowing only a random allocation procedure to influence treatment assignments, as shown in Figure 6B. Random procedures are denoted by circles in Figure 6. Figure 6B represents the data-generating process in RCTs defined by random treatment allocation. In the traditional frequentist approach, random allocation licenses the use of measures of uncertainty such as SEs and CIs for comparative causal estimates, such as the relative treatment effect shown in Figure 6B. Measures of relative, also known as comparative, treatment effects for survival outcomes include estimands such as HRs, differences in median or mean survival, or risk reduction at specified milestone time points (Table 1) [1].

A random sampling method aims to remove selection bias by obtaining a representative random subset of a patient population (Figure 6C), whereas random allocation uses a method, such as flipping a coin, to randomly assign treatments to patients in a sample (Figure 6B). We distinguish here “selection bias”, attributable to selective inclusion in the data pool due to sampling biases, from “confounding by indication” due to selective choice of treatment, which can be addressed by random treatment assignment [84,85,86]. 

The conventional statistical paradigm relies on the assumption that a sample accurately represents the population. For example, a simple random sample (SRS) of size 200 is obtained in such a way that all possible sets of 200 objects from the population are equally likely to comprise the sample. As a simple toy example, the 6 possible subsets of size 2 from a population of 4 objects {a, b, c, d} are {a, b}, {a, c}, {a, d}, {b, c}, {b, d} and {c, d}, so an SRS of size 2 is one of these 6 pairs, each with a probability of 1/6. Random sampling is used in sampling theory, whereas random allocation is used in the design of experiments such as RCTs [6,7,8]. They are connected by the causal principle that random procedures yield specific physical independencies; random allocation removes all other arrows towards treatment assignment (Figure 6B), while random sampling removes the arrow toward the selection node, S (Figure 6C) [44]. Accordingly, random allocation connects inferential statistics with causal parameters expressed as comparative estimands, such as between-treatment effects measured by ratios or differences in parameters (Table 1). Conversely, random sampling allows us to apply statistical inferences based on the sample to the entire population [87]. 

There exist some scenarios outside of medicine whereby both random sampling and random allocation are feasible (Figure 6D). One such example in the social sciences is to randomly sample a voter list from a population of interest and then randomly assign each voter to receive or not receive voter turnout encouragement mail. However, random sampling from the patient population for whom an approved treatment will be medically indicated is impossible in a clinical trial. A trial includes only subjects who meet enrollment criteria and enroll in the trial. Thus, they comprise a *convenience sample*, as denoted by the arrow toward the selection node, S, in Figure 6A,B, subject to a protocol’s entry criteria as well as other considerations such as access to the trial and willingness to consent to trial entry. Furthermore, patients are accrued sequentially over time in a trial. Due to newly diagnosed patients entering and treating patients leaving a population as they are cured or die, as well as changes in available treatments, any patient population itself constantly changes over time. Consequently, a trial’s sample is very unlikely to be a random sample that represents any definable future patient population. Even when inferences based on a trial’s data are reasonable, they may not be valid for a population because they only represent the trial’s convenience sample and not a well-defined patient population that will exist after the trial’s completion. Despite these caveats, data from an RCT can be very useful as a guide for future medical decision-making if a treatment difference is *transportable* from an RCT to a population-based on causal considerations, as extensively reviewed elsewhere [2]. 

## 10. Comparative and Group-Specific Inferences

Because each treatment group in most RCTs is a subsample of a convenience sample, one cannot reliably estimate valid SEs, CIs, *p*-values, or other measures of uncertainty for the outcomes within each treatment group, individual patient, or other subgroup. For such outcomes, only measures of variability such as the SD and IQR are useful [87,88,89]. However, this fact often goes unrecognized in contemporary RCTs, and within-arm statistics are reported that are of little inferential use for an identifiable patient population because they do not represent the population. For example, KEYNOTE-564 appropriately reported treatment comparisons in terms of the point estimate of HR, CIs, and a *p*-value for the DFS endpoint, but also provided the 95% CI for the proportion of patients who remained alive and recurrence-free at 24 months within each of the pembrolizumab and control groups [54]. Similarly, the CheckMate-214 phase 3 RCT of the new immunotherapy regimen nivolumab + ipilimumab versus the control therapy sunitinib in patients with metastatic ccRCC reported the 95% CI for the median OS outcomes of each treatment group. Nevertheless, it is important to note that these treatment-specific estimates cannot be reliably used to infer the treatment-specific OS outcomes of patient populations [90,91].

**Figure 7:**
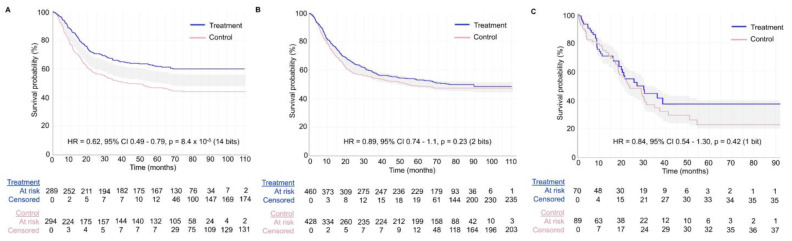
Example Kaplan–Meier survival plots from three hypothetical RCTs. The shaded gray area represents the midpoint of the treatment and control group survival estimates plus or minus the half-width of the 95% CI for the difference of each group’s Kaplan–Meier probability estimates. This gray polygon is centered at the midpoint between the two groups so that if it crosses one survival curve, it will also cross the other. It thus indicates that *p* > 0.05 (not multiplicity adjusted) for the null hypothesis of no treatment group difference in that time point, at time points where the gray polygon crosses the survival curves. HRs and their CIs and *p*-values were estimated using a univariable Cox proportional hazards model. (**A**) Example RCT with consistent signal of survival difference between the treatment and control (*p* < 0.05, corresponding to at least 4 bits of information against the null hypothesis). The corresponding Cox regression model yielded 14 bits of refutational information against the null hypothesis of no difference under the assumption that all other background model assumptions are correct. (**B**) Example RCT with no strong survival difference signal between the treatment and control groups, as indicated by the gray area consistently crossing the survival curves. The consistently narrow width of the gray polygon indicates that the trial results are compatible at the 0.05 level with no clinically meaningful difference between the treatment and control groups throughout the study. This is supported by the corresponding Cox model, which wielded only 2 bits of refutational information against the null hypothesis, as well as a 95% CI compatible with HR effect sizes ranging from 0.74, favoring the treatment group, to 1.1, favoring the control group. (**C**) This example RCT also has no strong survival difference signal between the treatment and control groups. The consistently wide gray area indicates that the signal is very low at all time points. Therefore, no inferences can be made on whether or not there is a treatment difference based on these survival curves. Accordingly, the corresponding Cox model yielded very low refutational information against the null hypothesis and a very wide 95% CI compatible with HR effect sizes as low as 0.54, strongly favoring the treatment group, and as high as 1.30, strongly favoring the control group.

Figure 7 shows the value of generating survival plots that properly focus on comparative inferences between treatment groups in RCTs. The survival plots were generated using the survplotp function from the rms package in R version 4.1.2 [92]. This function allows users to produce such plots in an interactive format that provides a real-time display of information, such as the number of patients censored and the number at risk per group at any time within the plot [93]. Furthermore, it allows one to visualize the time points where the *p*-value for the differences between groups is <0.05, denoted by the shaded gray area not crossing the survival curves. This shaded gray area represents the cumulative event curve difference. When it intersects with the survival curves, the *p*-value is >0.05. Narrow-shaded gray areas indicate less uncertainty, whereas wide-shaded gray areas represent high uncertainty in estimating the differences between groups. Figure 7A shows an example of an RCT with a consistent signal of an average survival difference between the treatment and control groups throughout the study, as also evidenced by the corresponding HR estimate of 0.62 with 95% CI 0.49–0.79 and *p*-value of 8.4 × 10^−5^, yielding 14 bits of refutational information against the null hypothesis. Figure 7B,C shows two RCTs with large *p*-values for the relative treatment effect measured by the HR estimate. However, the RCT in Figure 7B shows a consistent signal of no meaningful effect size difference between the treatment and control groups, as can be determined by looking at the consistently narrow cumulative event curve difference represented by the shaded gray area. The RCTs in Figure 7A,B yielded informative signals as evidenced by the narrow cumulative event curve difference. Conversely, Figure 7C shows the results of an uninformative RCT. This low signal is evident by the wide cumulative event curve difference throughout the survival plot and is consistent with the wide 95% CIs of 0.54–1.30 for the HR estimate. Therefore, no inferences can be made at any time point for the survival curves presented in Figure 7C. Readers inspecting the noisy data in Figure 7C may mistakenly conclude that there exists a signal of a survival difference favoring the treatment over the control group at the tail end of the curve from approximately 40 months onward. However, the wide cumulative event curve difference shows that the estimated curves from that time point onward are based almost exclusively on noise. This important visual information would be missed in survival plots that do not show the comparative uncertainty estimates for the differences between the RCT groups. In general, any Kaplan–Meier estimate becomes progressively less precise over time as the numbers at risk decrease, and at the tail end of the curve, it provides a much less reliable estimate due to the low numbers of patients followed at those time points. Indeed, only 21/159 = 13.2% of patients in the RCT shown in Figure 7C were in the risk set at 40 months. It has been proposed, accordingly, to refrain from presenting survival plots after the time point where only around 10% to 20% of patients remain at risk of the failure event [94]. A key point is that if we are to make decisions regarding a test hypothesis, such as the null hypothesis, then the binary decision to either reject or accept is inadequate because it cannot distinguish between the two very different scenarios shown in Figure 7B,C. Instead, we can more appropriately use the trinary of “reject” (Figure 7A), “accept” (Figure 7B), or “inconclusive” (Figure 7C). 

The number needed to treat (NNT), defined as the reciprocal of the estimated risk difference at a specified milestone time point, is a controversial comparative statistic originally proposed by clinicians to quantify differences between treatment groups in RCTs [95]. However, NNT is highly problematic statistically because values from the same RCT can vary widely for each milestone time point and follow-up time. Therefore, no single NNT can be used to comprehensively describe the results of an RCT. Additionally, NNTs are typically presented as point estimates without uncertainty measures such as CIs, thus creating the false impression that they represent fixed single numerical summaries [96,97,98,99]. Standard metrics, such as one-year risk reduction and its corresponding CI, are typically more reliable and interpretable summaries of RCTs. When NNTs are presented, their uncertainty intervals and the assumptions behind estimating this measure should be noted. 

## 11. Blocking and Stratification

Due to the play of chance, random sampling and random allocation both generate random imbalances in the distributions of patient characteristics between treatment arms. These imbalances are a natural consequence of random procedures, and uncertainty measures such as CIs account for such imbalances [8]. Complete randomization cannot perfectly balance baseline covariates between the treatment groups enrolled in an RCT [8,89,100,101]. The convention of summarizing covariate distributions by treatment arm and testing for between-arm differences, often presented in Table 1 of RCT reports, reflects nothing more than sample variability in baseline covariates between the groups, and has been called the “Table 1 fallacy” [63,102]. 

**Figure 8:**
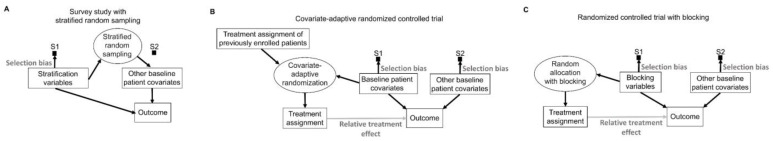
Selection diagrams distinguishing the causal effects of stratification, covariate-adaptive randomization, and blocking. (**A**) Surveys can obtain samples from explicitly specified stratification variables, which divide the population into smaller subgroups called “strata”. This induces a selection bias specifically for the stratification variables. Patients are then selected randomly from each stratum to form the final sample. (**B**) Clinical trials can ensure balance of specific baseline patient covariates by choosing the treatment assignment of each patient after adaptively accounting for their baseline patient covariates and for the treatment assignment of previously enrolled patients. Minimization is the most commonly used covariate-adaptive randomization method used in clinical trials. This covariate-adaptive “randomization” is actually a largely nonrandom treatment allocation method because it is influenced by the characteristics of earlier patients along with the baseline covariates of the current patient. (**C**) RCTs can limit the random allocation of treatments in such a way that each treatment group is balanced with respect to explicitly specified blocking variables, reducing the heterogeneity of the outcome. An additional non-mutually exclusive strategy would be to covariate adjust in the statistical model for the effect of the blocking variables on the outcome.

To mitigate potential covariate imbalances in survey studies used in sampling theory, the target population of patients can be partitioned into subgroups known as “strata”, based on specific covariates such as age, sex, race, or ethnicity, known as “stratification variables” [17]. By design, this sampling procedure induces a known selection bias for the stratification variables, which are sampled according to a specifically selected proportion, typically the population proportion, without deviations due to randomness [17,85,103]. The final sample then is formed by randomly sampling patients from each stratum, thus ensuring that there is no systematic selection bias for the non-stratified covariates (Figure 8A). For example, if a patient population has a 60% poor prognosis and 40% good prognosis, then a stratified sample of size 200 would consist of a random sample of size 120 from the poor prognosis subpopulation and a random sample of size 80 from the good prognosis subpopulation. There are numerous ways to obtain such representative samples, depending on the structure of the population of interest, particularly for unconscious units, such as sampling to ensure the quality of drug products in the market.

In experimental studies such as RCTs, covariates can be used adaptively during a trial to allocate treatment in a way that minimizes imbalances (Figure 8B) [104]. “Minimization” is the most commonly used of these covariate-adaptive randomization schemes [105]. Treatment allocation in such trials is largely nonrandom because it is directly influenced by the characteristics of earlier patients, along with the baseline covariates of the newly enrolled patients (Figure 8B) [101,106,107]. Thus, it is critical to choose appropriate statistical methods to validly analyze trials that use covariate-adaptive randomization methods [107]. Permutation tests can be used as the primary statistical analysis of the comparative treatment effect in RCTs that use covariate-adaptive randomization instead of conventional random treatment allocation [108]. While the balance achieved by covariate-adaptive randomization can potentially increase power compared with conventional RCTs, knowledge of the characteristics of earlier patients can allow trialists and other stakeholders to predict the next allocation, which increases the vulnerability of the trial to potential manipulation [101,108]. 

To avoid problems caused by the adaptive use of covariates to achieve balance during an RCT, imbalances can be prevented by a procedure known as “blocking” that deliberately restricts random allocation so that each treatment group is balanced with respect to prespecified “blocking variables” (Figure 8C). For example, in the KEYNOTE-564 phase 3 RCT, the primary outcome of disease recurrence or death was less likely in patients with stage M0 disease, defined as no history of radiologically visible metastasis, compared with patients who previously had such metastasis, classified as stage M1 with no evidence of disease (M1 NED) [54]. Therefore, to balance this variable between the group of patients randomized to adjuvant pembrolizumab and those randomized to placebo control, blocking was performed according to metastatic status (M0 vs. M1 NED). Within the subpopulation of patients with M0 disease, it was deemed that Eastern Cooperative Oncology Group (ECOG) performance status score and geographic location (United States vs. outside the United States) were baseline covariates that could meaningfully influence the survival endpoints. Accordingly, randomization was further blocked within the M0 subpopulation to balance the ECOG performance status and geographic location of patients randomized to adjuvant pembrolizumab or placebo control [54]. 

Medical RCTs often interchangeably use terms such as “blocking” and “stratification” [109]. However, stratification is a procedure used during sampling in a survey, whereas blocking is used during treatment allocation in an experiment. Conceptually, there is an isomorphism between random sampling, random allocation, and their respective theories and methods in the sense that they are logically equivalent, and thus one can be translated into the other [36,87,110]. Thus, random sampling can be viewed as a random allocation of patients to be included or excluded from a sample. Similarly, random allocation can be viewed as random sampling from the set of patients enrolled in either treatment group. To facilitate conceptual clarity, it may be preferable to keep the terminologies of sampling theory and experimental design distinct, given that each focuses on different physical operations and study designs (Table 2). Medical RCTs that do use the term “stratification” typically allude to the procedure whereby the prognostic variables (blocking variables) of interest are used to define “strata”, followed by blocking to achieve balance within each stratum [8,109].

Including blocking for metastatic status, ECOG performance status, and geographic location in the design of the KEYNOTE-564 RCT ensures that these variables will be balanced between the adjuvant pembrolizumab and placebo control groups. However, it is more efficient to also inform the statistical analysis model that one is interested in “apples-to-apples” comparisons between patients balanced for these blocking variables. To achieve this, the statistical model should adjust for these blocking variables [89,101,111]. This yields “adjusted” HRs that prioritize the comparison of patients randomized to adjuvant pembrolizumab with those randomized to placebo that had the same metastatic status, performance status, and geographic location [112]. Indeed, the statistical analysis models of KEYNOTE-564 adjusted for these blocking variables [54]. Of note, while blocked randomization and adjustment can prevent random imbalances of the blocking variables, they do not guarantee the balance of unblocked variables [113]. 

## 12. Forward and Reverse Causal Inference

Suppose that a patient with uncontrolled hypertension starts taking an investigational therapy, and two weeks later her blood pressure is measured and found to be within the normal range. This observed outcome under the investigational treatment is referred to as the “factual” outcome [114]. One may imagine the two-week outcome that would have been observed if the patient had received standard therapy instead. This may be called the “counterfactual” outcome since it was not observed [9,115,116]. Before a patient’s treatment is chosen and administered, the factual and counterfactual outcomes are called “potential” outcomes because both are possible, but they only become factual and counterfactual once a treatment is given. This structure provides a basis for a “reverse” causal inference task that aims to answer the question, “Did the intervention cause the observed outcome for this particular patient?” [9,115,117]. One may want to transport this knowledge to make predictions about the potential outcomes of using the investigational or standard therapy on other patients belonging to the same or different populations [2,118]. Such predictions require more assumptions than the reverse causal inference of RCTs, including causal assumptions about the transportability of the previously estimated relative treatment effects [2,80,81,119]. These “forward” causal inference models study the effects of causes to answer the question, “What would be the outcome of the intervention?”

To think about the forward causal effect of treatment X on an outcome Y, such as an indicator of response or survival time, one may perform the following thought experiment. To compare two treatments, denoted by 0 and 1, make two physical copies of a patient, treat one with X = 0 and the other with X = 1, and observe the two *potential outcomes*, Y(0) and Y(1). The difference Y(1) − Y(0) is called the *causal effect* of X on Y for the patient. Since this experiment is impossible, one cannot observe both potential outcomes. This is the central problem of causal inference [115]. Under reasonable assumptions, however, it can be proven that, if one randomizes actual patients between X = 0 and X = 1, producing sample means as estimators of the population mean treatment effects μ_0_ and μ_1_, then the difference between the sample means is an unbiased estimator of the between-treatment effect Δ = μ_1_ − μ_0_ for the population to which the sample corresponds. For example, if Y indicates response, then the sample average treatment effect is the difference between the two treatments’ estimated response probabilities, and the difference between the sample response probabilities follows a probability distribution with mean Δ; that is, it is unbiased. Assume that h_1_ is the hazard function (event rate) for the treatment group and h_0_ is the hazard function for the control group as previously described [1,2]. For HR = h_1_/h_0_, the relative treatment effect may be written as Δ = log(HR) = log(h_1_) − log(h_0_), and the sample log(HR) provides an unbiased estimator of Δ. The key assumptions to ensure this is that (1) whichever treatment, X = 0 or 1, is given to a patient, the observed outcome must equal the potential outcome, Y = Y(X); (2) given any patient covariates, treatment choice is conditionally independent of the future potential outcomes (that is, one cannot see into the future); and (3) both treatments must be possible for the patient. In terms of a DAG, (Figure 6B) [87], randomization removes any arrows from observed or unknown variables to treatment X, so the causal effect of X on Y cannot be confounded with the effects of any other variables. In particular, randomization removes the treatment decision from the physician or the patient, who would otherwise use the patient’s covariates or preferences to choose treatments (Figure 6A). An additional statistical tool is the central limit theorem (CLT), which says that, for a sufficiently large sample size, the distribution of the sample estimator is approximately normal with mean Δ and specified variance. This may be used to test hypotheses and compute uncertainty measures such as confidence intervals and *p*-values [120]. 

## 13. Generalizability and Transportability of Causal Effects

The term “generalizability” refers to the extension of inferences from an RCT to a patient population that coincides with, or is a subset of, a trial-eligible patient population [121,122,123]. The practical question is how a practicing physician may use inferences based on trial data to make treatment decisions for patients whom the physician sees in a clinic. Generalizability is the primary focus of sampling theory, as discussed above, and random sampling allows one to make inferences about broader populations. However, random sampling often is not practically feasible in trials, and clinical trial samples therefore are not representative. Thus, other sampling mechanisms have been proposed to facilitate generalizability, including the purposive selection of representative patients, pragmatic trials, and stratified sampling based on patient covariates [121].

The primary focus of experimental design is internal validity, which provides a scientific basis for making causal inferences about the effects of experimental interventions by controlling for bias and random variation [8,112,121,124]. The tight internal control exercised by experimental designs, such as RCTs, may make it difficult to use sampling theory to identify a population that the enrolled patient sample represents. The approach typically used to move causal inferences from an RCT to a population of patients, such as those seen in clinical practice, is called “transportability” [2,80,81,125]. Transportability relies on the assumption that the patients enrolled in a RCT and the target populations of interest share key biological or other causal mechanisms that influence the treatment effect. The key transportability assumption is that, while the individual treatment effects μ_0_ and μ_1_, such as mean survival times, may differ between the sample’s actual population and the target population, the between-treatment effect Δ = μ_1_ − μ_0_ is the same for the two populations. Transportability from experimental subjects to future patients seen in the clinic who share relevant mechanistic causal properties is a standard scientific assumption [2,80,81,82]. For example, inferences from an RCT comparing therapies that target human epidermal growth factor receptor 2 (HER2) signaling in breast cancer may be transported if a patient seen in the clinic has breast cancer driven by HER2 signaling, despite the fact that the clinic population is otherwise completely separate in space and time from the sample enrolled in the RCT [2,82].

External validity is the ability to extend inferences from a sample to a population, and thus it encompasses both generalizability and transportability [8,112,121,124]. In studies based on sampling theory, such as health surveys, external validity is mainly based on generalizability, i.e., whether the sample in the study is representative of the broader population of interest. In experimental studies, such as RCTs, external validity is predominantly based on transportability, i.e., whether the RCT investigated causal mechanisms that are shared with the populations of interest. Internal validity, however, is the more fundamental consideration in both sampling theory and experimental design. External validity is meaningless for studies without internal validity.

## 14. Representativeness and Inclusiveness

Because experimental design focuses on making internally valid causal inferences, it has been argued in both the statistical and epidemiological literature that sampling representativeness is incongruous with the goal of experiments such as RCTs [8,126,127,128,129]. For example, when we evaluate a new cancer therapy preclinically in mice, we do not randomly select a representative sample of mice to be included in the study. Instead, we choose a homogeneous group of mice that closely recapitulates the biological mechanisms we wish to study. This aspect of experimental design is independent of whether the treatment is randomly allocated among the mice, and it corresponds to controlling for the effects of “Baseline patient covariates” on “Outcome” in Figure 6. To precisely estimate a between-treatment effect in an RCT, the effects of baseline patient covariates on the outcome may be controlled by balancing on them in the randomization, e.g., by stratification and blocking [8,82]. For example, in an RCT comparing two therapies that target HER2 signaling in breast cancer, it may be more efficient to restrict enrollment to a sample of HER2-positive patients [2]. In contrast, if there is a reasonable possibility of treatment effects not mediated by HER2 signaling, HER2-negative patients may also be enrolled, with randomization that balances within the HER2-positive and -negative subgroups. The statistical model of analyses, prespecified in the experimental design, can then allow for different between-treatment effects in the two HER2 subgroups. Similarly, when there is a mechanistic rationale for investigating interactions between treatment and certain baseline patient covariates, such as sex or race, statistical inferences should be based on a prespecified regression model that includes such interactions [2,8,130]. For example, inferences may consider different between-treatment effects for male and female patients. A potential major issue is that investigating causal interactions between baseline patient covariates or subgroups and treatments requires a larger overall sample size to make reliable inferences [8,57,131]. 

The fact that representativeness is not necessary to make comparative inferences in RCTs does not invalidate ethical and societal considerations for inclusiveness, which is distinct from representativeness. Inclusiveness is the goal of increasing the participation of minority and/or underserved populations in RCTs to reduce healthcare disparities [132,133,134]. This is different from the scientific goal of representativeness used in sampling theory studies, such as health surveys [135,136]. An important scientific issue that may motivate inclusiveness is the question of whether the magnitude of a causal between-treatment effect may differ between minority subgroups defined, for example, by race or gender. If such a treatment-subgroup interaction is suggested mechanistically by biological knowledge or statistically by historical data, and if the difference in magnitude is large enough to change inferences regarding the comparative treatment effect, then not including a sufficient number of minority patients in an RCT serves society poorly. 

## 15. Relevance and Robustness

A major goal of RCTs is to generate knowledge that can inform physicians in making inferences and decisions for the patients seen in their clinic [1,2]. For example, suppose the goal is to use the results of the KEYNOTE-564 phase 3 RCT, which compared adjuvant pembrolizumab to placebo (surveillance) in patients with ccRCC in terms of survival, to make a treatment choice for a patient with ccRCC seen in the clinic [2,54,137]. This requires accounting for how treatment and a patient’s baseline covariates affect their survival (Figure 6). Patient relevance refers to how well a statistical regression model accounts for attributes of a specific patient seen in the clinic, to generate tailored estimates of survival or other clinical outcomes [68,138]. An ideal scenario is for an RCT to perform an “apples-to-apples” comparison of the effect of adjuvant pembrolizumab versus placebo between RCT patients with baseline covariates identical to those of a patient in the clinic. Perfect patient relevance would require a statistical model to account for every aspect of a patient’s biology, environment, and other covariates that can influence the outcome of interest. However, this is an unrealistic goal, and the relevance of a statistical model must be balanced with robustness and practicality [3,4,68,138,139]. 

Robustness implies that inferences will be valid for a wide range of different patient covariates. The higher the robustness of RCT results, the more applicable they are for making inferences across a heterogeneous patient population [68,138,140]. Robustness generally describes the extent to which results can be reproduced after altering experimental conditions. For example, during preclinical assessment of an investigational cancer therapy, the robustness of causal inferences is increased if they can be replicated qualitatively with a different cell line or animal model. Reproducibility is a distinct concept that describes whether the results of an experiment can be obtained, possibly with small random variation, after repeating the experiment under identical conditions [140].

## 16. Intention to Treat and Per Protocol

**Figure 9:**
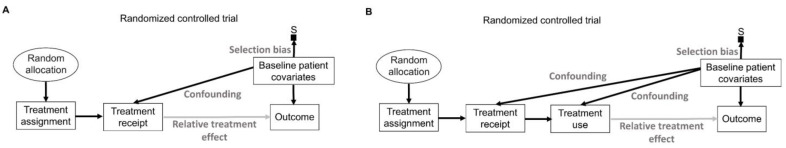
Selection diagrams distinguishing per intention to treat (ITT), per protocol (PP), and as treated (AT) in RCTs. (**A**) Diagram illustrating the scenario whereby patients randomly assigned to a treatment did not always receive it. The relative treatment effect parameter from the PP analysis is more relevant for direct patient care but is susceptible to confounding biases from covariates that may have influenced treatment receipt. (**B**) Diagram illustrating the scenario whereby patients randomly assigned to a treatment did not always receive it, and those that received it did not always use it. The relative treatment effect parameter from the AT analysis is more relevant for direct patient care but is susceptible to confounding biases from covariates that may have influenced treatment receipt and treatment use.

While inanimate units, such as plots of land in agricultural experiments, will always follow the allocated intervention in an experiment, experimental design and analysis of RCTs in medicine are more complex because patients may not always follow the randomly assigned treatment (Figure 9A) [141,142,143]. “Intention-to-treat” (ITT) analyses estimate the relative treatment effect for patients based on their treatment assignment, regardless of whether they actually received the assigned therapy. Uncertainty measures for the relative treatment effect generated by ITT analyses of RCT data are justifiable by the random allocation procedure (Figure 9A). However, because the actual treatment received is the source of biological efficacy, clinicians are typically interested in predicting the potential outcomes if their patient actually receives a particular therapy. Corresponding causal RCT parameters for such inferences are derived from “per-protocol” (PP) analyses that estimate the relative treatment effect for the therapies that patients actually received [143]. However, as shown in Figure 9A, random treatment assignment removes all systematic confounding influences on the assigned treatment but does not prevent the potential influence of patient covariates on whether the treatment was actually received. This means that PP analysis models should account for possible confounding biases to reliably estimate the relative treatment effect of the treatment received on the outcome of interest. This can be facilitated by recognizing that “Treatment assignment” in Figure 9A is an instrumental variable for the relative treatment effect of the treatment received on the outcome. Instrumental variable methodologies developed in econometrics and epidemiology can be used to account for the systematic confounding influence of the treatments received in RCTs [142]. A complementary strategy is to enforce RCT internal validity by carefully designing, implementing, and monitoring the trial so that the treatment received corresponds to the treatment assignment as much as possible and is not influenced by patient covariates.

In a recent RCT, patients were randomly allocated to receive an invitation to undergo a single screening colonoscopy or to receive no invitation or screening [144]. The ITT analysis, termed “intention-to-screen” by the study, found that the risk of colorectal cancer at 10 years was reduced in the invited group compared with the group randomly allocated to no invitation (the usual-care group) with risk ratio (RR) = 0.82, 95% CI 0.70–0.93, and *p* ≈ 0.006, corresponding to 7 bits of refutational information against the null hypothesis of no difference in colorectal cancer risk at 10 years. However, only 42% of invited patients actually underwent colonoscopy [144]. Thus, the estimate yielded by the ITT analysis is more relevant for forward causal inferences related to implementing a health policy of screening colonoscopy invitation. On the other hand, the estimate of higher interest to clinicians and patients is how much an actual screening colonoscopy can modify the risk of colorectal cancer at 10 years. This was provided by the adjusted PP analysis, which reported RR = 0.69, 95% CI 0.55–0.83, and *p* ≈ 0.0005, corresponding to 11 bits of refutational information against the null hypothesis. The caveat is that, although the PP analysis is more relevant to direct patient care, its estimates rely on additional assumptions, reviewed elsewhere [143,145], and are less physically justifiable from the random allocation than those from the ITT analysis. For simplicity, we have assumed here that what the authors used in their ITT analysis fully corresponded to the random allocation. However, some patients allocated to each group actually were excluded, died, or were diagnosed with colorectal cancer before being included in the study and were thus excluded from the ITT analysis [144].

An additional distinction, often used in RCTs of medical devices, separates the PP analysis of those who received the treatment from the “as-treated” (AT) analysis of those who actually used their assigned treatment (Figure 9B). In these scenarios, the AT relative treatment effect estimate is the most relevant for clinical inferences but again requires careful modeling of potential systematic confounders (Figure 9B). In the colonoscopy RCT [144], ITT would analyze patients as per their assigned screening intervention regardless of whether the assigned screening invitation was actually sent to the patients, PP would analyze patients based on whether or not they received the assigned invitation, regardless of whether they actually underwent colonoscopy, and AT would analyze patients based on whether they actually underwent colonoscopy, regardless of whether they were originally randomly assigned to the colonoscopy or whether they received an invitation to undergo colonoscopy.

## 17. Prognostic and Predictive Effects

**Figure 10:**
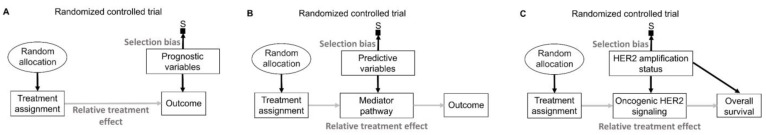
Selection diagrams representing the data-generating processes of prognostic and predictive effects in RCTs. (**A**) Prognostic biomarkers are baseline patient variables that directly influence the outcome and not the relative treatment effect. Thus, relative treatment effect parameters such as HRs and odds ratios (ORs) are assumed to be stable for all patients in the RCT cohort. (**B**) Predictive biomarkers are baseline patient variables that influence the relative treatment effect via their effect on the mediator pathway that transmits the effect of treatment assignment on the RCT outcome. HRs, ORs, and other relative treatment effect parameters can change depending on the values of the predictive biomarker. (**C**) In patients with breast cancer, HER2 amplification status acts as both a prognostic and predictive biomarker.

In addition to the effect of the assigned treatment on the outcomes observed in RCTs, baseline patient covariates, also known as moderator variables, may affect the magnitude and direction of the treatment effect [2,146]. These moderator effects can be distinguished based on the two underlying data-generating processes represented in Figure 10. The first type (Figure 10A) has been described in the literature using various terms such as “risk magnification”, “risk modeling”, “effect measure modification”, “additive effect”, “main effect”, “heterogeneity of effect”, or “prognostic effect” [1,2,112,146,147,148]. The second type (Figure 10B) has been described as “biologic interaction”, “effect modeling”, “treatment interaction”, “multiplicative effect”, “biological treatment effect modification”, or “predictive effect” [1,2,112,146,147,148]. For simplicity, we will adopt the terms “prognostic” and “predictive”, often used in medical RCTs, to distinguish between the two moderator effect types. 

In an RCT, prognostic variables may directly affect the outcome of interest but do not interact with any treatment. Consequently, they do not affect the comparative treatment effect parameter, such as an HR, which remains stable across patients (Figure 10A). In contrast, a variable that is predictive for a particular treatment changes the relative treatment effect in RCTs by acting on pathways that mediate the effect of the assigned treatment on the outcome (Figure 10B). Thus, the HR for survival in an RCT may differ between subgroups of patients harboring distinct values of a predictive biomarker. Predictive biomarkers often have direct prognostic effects as well. For example, patients with breast cancer harboring amplifications of the *HER2* gene, found in 25% to 30% of breast cancers [149,150], have different prognoses than patients without such *HER2* amplifications [151], regardless of what treatment is given. The targeted agent trastuzumab was developed to specifically target the oncogenic HER2 signaling that drives the growth of *HER2*-amplified breast cancers [152]. Therefore, in an RCT comparing the use of trastuzumab versus placebo, *HER2* amplification status acts as both a prognostic biomarker that directly influences patient survival and a predictive biomarker that influences the relative treatment effect for trastuzumab (Figure 10C). Patients without *HER2* amplification in their tumors would be expected to derive no benefit from trastuzumab [2,152].

Nuisance variables are defined as variables that are not of primary interest in a study but still must be accounted for because they may influence the heterogeneity of the outcome of interest. Prognostic variables may act as nuisance variables in RCTs [8,99]. Variables expected to have the strongest prognostic effects on the outcome of interest should be used as blocking variables in RCTs (Figure 8C). The primary endpoint analyses of randomized block designs will model the prognostic effects but rarely the predictive effects of blocking variables [8,89]. This is because the predictive effects of patient covariates on the relative treatment effect require large enough replicates (hence, large sample sizes) to be estimated reliably in RCTs [8,57,131]. For this reason, predictive biomarkers typically are first identified in exploratory analyses and characterized in preclinical laboratory studies, with subsequent biologically informed RCTs specifically enriching for patients with these biomarkers [2,82,153,154]. Modern RCT designs may also attempt to adaptively enrich such biomarkers during trial conduct based on interim analyses of treatment response and survival times [154,155].

Due to their powerful direct effects on patient outcomes, prognostic biomarkers should always be considered when making patient-specific clinical inferences and decisions [1,148]. On the other hand, identifying predictive effects during an RCT carries the risk of misleading inferences and thus should be performed very rigorously [156]. For example, an exploratory analysis of the COSMIC-313 RCT investigated whether the International Metastatic Renal Cell Carcinoma Database Consortium (IMDC) risk score [157] can be used as a predictive covariate for the relative treatment effect of the cabozantinib + nivolumab + ipilimumab triplet therapy versus placebo + nivolumab + ipilimumab control [64]. Similar to the example shown in Figure 7A, the Kaplan–Meier survival curves for the IMDC intermediate-risk subgroup showed a clear signal of relative treatment effect difference for PFS favoring the triplet therapy over the control based on a total of 182 PFS events. The HR for PFS was 0.63 with 95% CI 0.47–0.85 and *p* ≈ 0.002, corresponding to approximately 9 bits of information against the null hypothesis. However, in the IMDC poor-risk subgroup there were only a total of 67 PFS events, yielding very noisy survival curves, similar to the example shown in Figure 7C. The HR estimate for PFS in the IMDC poor-risk subgroup was 1.04 with 95% CI 0.65–1.69 and *p* ≈ 0.88, corresponding to 0 bits of information against the null hypothesis. Thus, no inferences can be made regarding the predictive effect of the IMDC intermediate- versus poor-risk subgroups in COSMIC-313 because only the intermediate-risk subgroup yielded precise estimates, whereas the poor-risk subgroup estimates were unreliable due to their imprecision. However, because the survival curves did not present the wide uncertainty intervals for the comparative difference between treatment groups as was executed in Figure 7C, it was incorrectly concluded that the RCT showed no difference in relative treatment effect for the IMDC poor-risk subgroup and thus that the triplet therapy should be favored only in the IMDC intermediate-risk subgroup. Such mistaken inferences from noisy results are very frequent when looking at outcomes within each risk subgroup [156]. To obtain clinically actionable signals, it is preferable instead to look for either prognostic or predictive effects in the full dataset of all patients enrolled in the RCT. Indeed, if we assume IMDC risk to be a prognostic biomarker in the full dataset, as indicated by the fact that COSMIC-313 used it as a blocking variable in its primary endpoint analysis, then patients with IMDC poor-risk disease will derive more absolute PFS benefit in terms of risk reduction at milestone time points than patients with IMDC intermediate-risk disease [1,63]. This is an example in which ignoring prognostic effects while hunting for predictive biomarkers, a type of data dredging, can lead to erroneous clinical inferences and decisions. 

**Figure 11:**
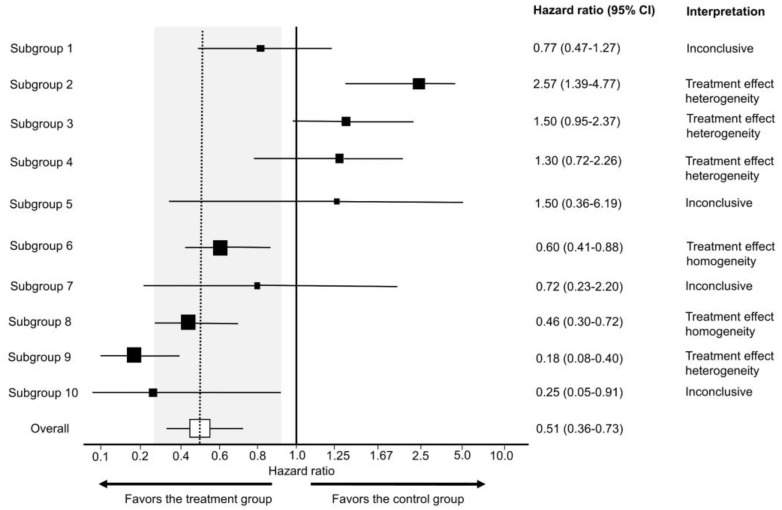
Example forest plot from a hypothetical RCT of an investigational treatment versus control. The forest plot is used to look for predictive effects expressed as differences in HR estimates in different subgroups compared with the overall RCT cohort. The dotted vertical line highlights the relative treatment effect point estimate for the overall cohort, also known as the main effect. The size of the black squares corresponds to the sample size of each subgroup. The white square represents the overall RCT cohort. The horizontal lines represent the 95% Cis. The shaded gray area represents the indifference zone for the HR estimate in the overall cohort, assuming that relative treatment effects between 80% and 125% of the 95% CI for the overall cohort do not represent clinically meaningful differences between each subgroup and the overall cohort. In this example, the 95% CI for the HR in the overall cohort is 0.36–0.73, corresponding to an indifference zone of 0.29–0.91. Therefore, treatment effect homogeneity is suggested for all subgroups with the 95% CI that are only compatible with values within the indifference zone (gray area). Treatment effect heterogeneity is suggested in subgroups with 95% CI that do not overlap with the dotted vertical line. All other subgroups are inconclusive.

Predictive effects are analyzed by including treatment–covariate interaction terms in the statistical regression model used to analyze the RCT dataset [2,19]. However, uncertainty measures such as *p*-values and CIs for these interaction effects are only physically justifiable in RCTs where both random sampling and random allocation are performed (Figure 6D). The vast majority of RCTs perform only random allocation, and therefore the uncertainty measures of treatment–covariate interaction terms are not linked to a physical randomization process, since patients were not randomized to their predictive covariates (Figure 6B). For this reason, modeling these interactions to look for predictive effects in RCT datasets is typically considered exploratory at best, and some journal guidelines specifically recommend against the presentation of *p*-values for predictive effects due to the substantial risk of misinterpretation [158]. However, the same journals also allow the presentation of a cruder visual tool called “forest plots” to perform graphical subgroup comparisons for predictive effects in RCTs [1,156,159]. Forest plots rely on the use of CIs for patient subgroups determined by their presumably predictive covariates (Figure 11). Neither these inferences nor *p*-values for interaction are physically justifiable due to the lack of random sampling in typical RCT designs. The use of forest plots in this way can easily lead to spurious inferences and may be considered data dredging.

Even when the Cis used in forest plots for subgroup comparisons are valid, the majority of these graphs do not provide clinicians with meaningful indications of patient heterogeneity in practical terms. Empirically, most subgroup analyses of RCTs for predictive effects using forest plots presented at the 2020 and 2021 Annual Meetings of the American Society of Clinical Oncology (ASCO) were found to be inconclusive, yielding no informative signals to either refute (treatment effect heterogeneity) or support (treatment effect homogeneity) the assumption that relative treatment effect parameters such as HRs are stable across subgroups [156]. All of these forest plots were based on results from a frequentist model, and only 24.2% included one or more subgroups suggestive of treatment effect heterogeneity [156]. Because clinicians often seek to determine evidence of treatment effect homogeneity from forest plots, a practical approach has been developed to estimate an “indifference zone” of no clinically meaningful difference for the relative treatment effect estimate between the overall RCT cohort and each subgroup visualized by a forest plot [156]. The assumptions and formulas to estimate the indifference zone are detailed by Hahn et al. [156], and a simple spreadsheet (Supplementary File S2) that can be used by clinicians to make these estimations is provided here. The indifference zone shown in Figure 11 uses the 80% to 125% bioequivalence limits commonly used by the World Health Organization and the FDA, and they correspond to the clinically non-inferior HR effect size interval of 0.80 to 1.25 typically used in RCTs [160]. Even after using this approach to maximize the information yielded by forest plots, 57.2% of subgroup comparisons presented in forest plots at the 2020 and 2021 annual ASCO meetings were inconclusive, 41.4% showed evidence of treatment effect homogeneity, and only 1.6% were suggestive of treatment effect heterogeneity [156].

Given these limitations of forest plots, analyses for identifying treatment effect heterogeneity should focus instead on prespecified biologically and clinically plausible predictive biomarkers. Moreover, forest plots often arbitrarily dichotomize subgroups, e.g., into patients aged younger or older than 65 years. Such arbitrary cutoffs misleadingly assume that all patients younger than 65 have the same expected outcome. Rather than arbitrarily categorizing covariates into subgroups, it is more reasonable to preserve all information from continuous variables and fully model treatment-covariate interaction while properly adjusting for other prognostic or predictive effects that can influence outcome heterogeneity [1,3,112,161]. For example, age-specific treatment inferences and decisions were identified via a utility-based decision analysis based on robust Bayesian nonparametric modeling of the data from the CALGB 40503 phase 3 RCT comparing letrozole alone versus letrozole + bevacizumab in hormone receptor-positive advanced breast cancer [3,162].

If forest plots of RCT subgroups are presented, then cautious interpretation should be promoted by journals, professional organizations, and regulatory bodies. Figure 11 provides teaching examples of how to interpret different subgroup patterns in forest plots. An example of how and why forest plots should be interpreted cautiously is provided by analyses of the POUT phase 3 RCT, which tested whether adjuvant chemotherapy improved outcomes compared with surveillance in patients with upper tract urothelial carcinoma (UTUC) [163]. The results for the primary endpoint of DFS showed an estimated HR of 0.45 favoring adjuvant chemotherapy with 95% CI 0.30–0.68 and *p* = 0.0001, corresponding to 13 bits of refutational information against the null hypothesis of no DFS difference between the two treatment groups. The study’s forest plot illustrating estimated differences in the HR for DFS among the blocking variables and tumor stage was correctly interpreted as inconclusive for any evidence of treatment effect heterogeneity [163]. However, a common mistake when interpreting forest plots is to conclude that the relative treatment effect estimate is not significant for subgroups with CIs that cross the vertical line corresponding to the null effect, i.e., 1.0 for ratios such as HRs, ORs, and RRs [1,39,156,164]. Such examples and their proper interpretation are shown in Subgroups 1, 4, 5, and 7 in Figure 11. The POUT forest plot included a subgroup comparison by lymph node involvement whereby N0 represented the patients without lymph node involvement by UTUC, and N+ were the patients who had lymph node-positive disease. The N0 subgroup, which included 236 patients and 82 events, yielded a clear DFS signal in favor of adjuvant chemotherapy with HR = 0.40, 95% CI 0.25–0.63, and *p* ≈ 0.0001, corresponding to 13 bits of refutational information against the null hypothesis. The relationship of this subgroup to the overall cohort is similar to that of Subgroup 8 in Figure 11. Conversely, the N+ subgroup, which included only 24 patients and 13 events, yielded inconclusive results with HR = 0.90, 95% CI 0.30–2.71, and *p* = 0.86, corresponding to zero bits of information against the null hypothesis, similar to the wide CIs of Subgroup 7 in Figure 11. The 80% to 125% indifference zone for the main effect corresponds to HRs between 0.24 and 0.85, so there is evidence of treatment effect homogeneity between the N0 subgroup and the overall effect, as expected since most patients in the POUT trial belonged to the N0 subgroup. 

Patients with N+ UTUC have a higher risk of disease recurrence or death at any time point compared with N0 UTUC patients. Thus, if this blocking variable is analyzed as prognostic, as was executed in the primary endpoint analysis model of the POUT trial [163], then adjuvant chemotherapy is more likely to yield higher milestone time point risk reduction for disease recurrence or death in patients with N+ compared with N0 UTUC. This is consistent with the clinical intuition that patients with higher stage N+ disease are more likely to derive benefit from adjuvant chemotherapy than those with lower stage N0 UTUC. However, clinicians scanning the POUT forest plot for predictive effects may erroneously conclude that the exact opposite is true; whereas there was a clear signal favoring adjuvant chemotherapy in the N0 subgroup, the CIs for the N+ subgroup crossed 1.0, which can be misinterpreted as evidence for no effect in the N+ subgroup.

## 18. Superiority and Noninferiority

The primary goal of RCTs is to investigate how likely it is that a new intervention is superior to the control by gathering data that can potentially refute the null hypothesis of no difference. Such superior RCTs may indeed yield precise results with narrow CIs that are compatible with large or small relative treatment effect sizes (e.g., Figure 7A vs. Figure 7B). In the latter case, we can conclude that the new intervention is not meaningfully different from the control. For example, the VALIANT RCT compared valsartan with captopril in patients with complicated myocardial infarction and reported an HR estimate for the death of 1.0 with 95% CI 0.91–1.08 and *p*-value = 0.98, corresponding to zero bits of information against the null hypothesis [165]. The large *p*-value indicates that there is little evidence that one treatment is superior to the other. More importantly, the narrow 95% CI was compatible at the 0.05 level (≤4 bits of refutational information), with HR = 0.91, favoring the valsartan group, and HR = 1.08, favoring the captopril group. These HR effect sizes suggest no clinically meaningful difference, as the standard, commonly accepted thresholds for clinical equivalence are HRs ranging from 0.8 to 1.25 or, more conservatively, 0.9 to 1.1 [156,166]. 

Superiority RCTs can also yield inconclusive results (Figure 7C). For example, an RCT testing remdesivir versus placebo for the treatment of severe COVID-19 reported an HR estimate for time to clinical deterioration of 0.95 with 95% CI 0.55–1.64 and *p* = 0.86, corresponding to zero bits of information against the null hypothesis of no difference [167]. In this case, however, the wide 95% CI was compatible with both HR = 0.55, strongly favoring remdesivir, and HR = 1.64, strongly favoring placebo. Both this and the VALIANT RCT were interpreted as showing “no statistically significant difference” due to the large *p*-values [165,167]. However, the results of the two RCTs were vastly different, as suggested by the different widths of their 95% CIs, as VALIANT was precise enough (narrow 95% CI) to demonstrate a lack of clinically meaningful difference, whereas no conclusions could be drawn from the remdesivir RCT due to the wide 95% CI. 

Noninferiority RCTs differ from superiority trials in that the tested hypothesis is not the null hypothesis of no difference but the hypothesis that the new intervention is worse than the control by more than a small “noninferiority margin” [168,169]. While both superiority and noninferiority RCTs can be used to demonstrate a lack of clinically meaningful difference, noninferiority RCTs are far more likely to yield a verdict of noninferiority and thus are considered a “safe design” likely to result in a “positive” publication [169,170,171]. The underlying reason is that, whereas the ITT analysis of superiority RCTs penalizes poor patient adherence to the randomly assigned intervention, the opposite is true for noninferiority trials [169]. For example, in a superiority RCT aiming to refute the null hypothesis of no difference between an investigational treatment and placebo, if too many patients randomly allocated to the new treatment are non-compliant then this reduces the chances of showing a difference between the groups in the ITT analysis because not enough patients will have been exposed to the new treatment to yield a clear signal. Conversely, if the same thing happens in a noninferiority RCT then this increases the chances of a biased conclusion of noninferiority between the groups in the ITT analysis [169]. Indeed, the frequency of reaching a conclusion of noninferiority in noninferiority RCTs has been found to exceed 80% [170,171]. For this reason, it is recommended to present both the ITT and PP analyses in noninferiority RCTs [169]. Discrepancies between the two results should prompt further careful interrogation of the dataset and trial conduct. In particular, close attention should be paid to patient adherence to the randomly allocated intervention and other aspects of internal validity, such as complete and rigorous follow-up, before drawing conclusions about noninferiority.

## 19. Enthusiastic and Skeptical Priors

As already discussed, the ability to specify a prior distribution that reflects current knowledge before collecting data from an RCT is a key feature of Bayesian models. Skeptical priors assume that treatment differences are unlikely. Conversely, enthusiastic priors assume that the treatment is better than the control in the RCT [172,173,174]. Skeptical and enthusiastic priors are particularly useful when considering whether to stop an RCT early after an interim analysis. The goal is to counterbalance prior opinions of those who would doubt the observed interim analysis results. In particular, enthusiastic priors can be used to stop an RCT early for futility. That is, a futility monitoring procedure with an enthusiastic prior stops a trial if interim data show strong evidence of futility. Otherwise, the trial is continued. For similar reasons, skeptical priors are appropriate when considering stopping an RCT early for efficacy [173]. If the trial proceeds to completion, it should have accumulated enough data to convince all subject matter experts of the presence or absence of a relative treatment effect, including pessimists using skeptical priors and optimists using enthusiastic priors.

An RCT that compared immediate venovenous extracorporeal membrane oxygenation (ECMO) versus conventional control treatment, which included delayed ECMO, in patients with severe acute respiratory distress syndrome (ARDS) [175] serves as an example in which a Bayesian analysis might have prevented an unreasonable interim analysis decision. The trial generated controversy because it was stopped early for futility by the data safety monitoring committee. The interim results did not reach the prespecified frequentist significance level despite yielding an HR estimate of 0.70 for death within 60 days after randomization with 95% CI 0.47–1.04 and *p*-value = 0.07, corresponding to 4 bits of refutational information against the null hypothesis favoring ECMO over the control, which is similar refutational information to that of the standard *p*-value threshold of 0.05 [175,176]. Subsequent post hoc interim analysis of the data with a Bayesian model using a moderately enthusiastic prior yielded a 99% posterior probability of a 60-day mortality benefit for ECMO compared with the control. The conclusion based on this posterior inference clearly shows that the trial should have continued [177]. A model with a noninformative beta (0.5,0.5) prior yielded a posterior probability of 94.8% that ECMO reduces 60-day mortality compared with the control [19], which is equivalent to 19 to 1 odds in favor of ECMO. While both frequentist and Bayesian analyses are consistent with an efficacy signal favoring ECMO, the inferences from the Bayesian analysis are more intuitive and demonstrate that the decision to stop the RCT for futility was based on unreasonable frequentist decision-theoretic trade-offs codified by prespecified type I and II error probabilities.

## 20. Intermediate Endpoints and Overall Survival

**Figure 12:**
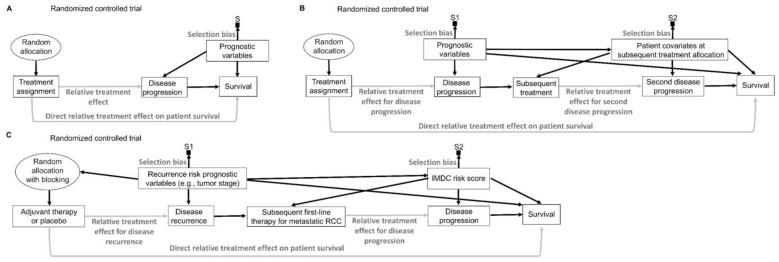
Selection diagrams representing the data-generating processes of clinical endpoints in RCTs. (**A**) In RCTs where no subsequent options are available, intermediate events such as disease progression will directly correlate with survival. Thus, the prognostic variables that influence disease progression will also influence survival directly or indirectly via the disease progression pathway. Blocking or adjusting for these variables will increase the reliability of disease progression and survival estimates. (**B**) In RCTs where subsequent therapies are available, random allocation removes all other causal influences on the treatment assignment of the first therapy, physically justifying the use of uncertainty estimates of the direct relative treatment effect on patient survival and the relative treatment effect for intermediate endpoints such as disease progression. These are the parameters used for intermediate survival endpoints such as PFS or DFS. However, the effect of the original treatment assignment on survival will also be mediated indirectly by subsequent therapies and disease progression events, which can be confounded by patient covariates at the time of subsequent treatment allocation. (**C**) Example RCT to evaluate the effect of adjuvant therapy or placebo in patients with localized ccRCC. Baseline prognostic factors, such as tumor stage, that influence disease recurrence can be balanced by blocking and adjusting in the statistical model to facilitate estimation of the DFS endpoint. However, upon disease recurrence, the choice of subsequent therapies will be influenced by covariates such as the International Metastatic Renal Cell Carcinoma Database Consortium (IMDC) risk score for metastatic RCC. This confounding influence and mediating effect of subsequent therapies and disease progression need to be modeled for reliable estimation of the OS endpoint.

Consideration of the data-generating processes (Figure 12) can help to determine the most appropriate endpoints and statistical analysis models for a RCT. Clinical endpoints are defined as outcomes that reflect how patients feel, function, or survive [178]. The International Council for Harmonization of Technical Requirements for Pharmaceuticals for Human Use (ICH) E9 recently issued the addendum R1 on “Estimands and Sensitivity Analyses in Clinical Trials”, whereby an estimand is defined as the parameter θ corresponding to the comparative relative treatment effect of the RCT interventions on the clinical endpoint of interest [179,180]. OS time is a clinically meaningful, intuitive, and objective clinical endpoint to compare the efficacy of a new intervention versus control in an RCT [181]. Intermediate endpoints are clinical endpoints such as DFS and PFS time, which themselves directly measure clinical benefit but do not necessarily reflect the end of a patient’s treatment course [178,181,182,183]. For example, the outcome represented by the time to recurrence (TTR) clinical endpoint in oncology RCTs is used to record the presence or absence of a cure. Those not cured of the cancer will experience the TTR event, whereas those cured will never experience this event. Conversely, patients not cured may die from other causes but not from their cancer, and thus improvements in cure rates may not be directly captured by long-term endpoints such as OS. Thus, the value of each clinical endpoint will be context dependent and patient-specific. 

Composite clinical endpoints that include the word “survival” in their name, such as DFS time, measure time to either the intermediate endpoint or death, whichever comes first. Those that do not include “survival” measure only the intermediate endpoint as an event. Thus, TTR only considers disease recurrence as an event, whereas DFS, also known as recurrence-free survival (RFS), considers either disease recurrence or death as an event. Similarly, time to progression (TTP) measures only the intermediate endpoint of disease progression, assuming that death without progression is an independent censoring event, whereas PFS accounts for either disease progression or death, whichever comes first [184]. A problem with endpoints such as TTR and TTP is that a patient’s death is considered simply as a noninformative censoring event. That is, the occurrence of death is assumed to be independent of the occurrence of the intermediate endpoint, e.g., disease progression, which may be untrue on fundamental grounds. On the other hand, a problem with composite outcomes such as PFS time is that death without recurrence or disease recurrence prior to death carries the same implication regarding the treatment effect. 

Surrogate endpoints are early or intermediate variables used in RCTs to make inferences about the effects of treatment on long-term outcomes, such as PFS or OS time [178]. A surrogate may be a biomarker, tumor response, or other endpoints that can be measured in a short timeframe, such as at the end of one cycle of therapy. An example is serum measurements of prostate-specific antigen (PSA) that may, in certain contexts, be used to predict PFS or OS [185]. Intermediate endpoints, such as PFS, also may be used as surrogates to predict final endpoints, such as OS. A common problem with any surrogate endpoint is that it is never perfectly associated with the long-term outcome of interest and may produce misleading inferences. An example was the use of complete response (CR) evaluated at 90 days post-transplant as a surrogate to the primary endpoint of PFS in a phase 2 RCT of patients with multiple myeloma randomized to either busulfan + melphalan or melphalan alone as the preparative regimen for autologous hemopoietic cell transplantation (auto-HCT) [19,186]. In the melphalan monotherapy control arm, 13/32 patients (40.6%) achieved 90-day CR compared with only 6/44 patients (13.6%) in the busulfan + melphalan combination arm. However, the combination of busulfan + melphalan yielded a longer estimated PFS compared with melphalan monotherapy, with HR = 0.53, 95% CI 0.30–0.91, and *p* = 0.022, corresponding to 6 bits of refutational information against the null hypothesis of no PFS difference between the two groups [19,186]. The divergent conclusions can be attributed to the fact that PFS is a more informative endpoint that takes into account the time to progression or death, whereas the former only considers a dichotomized outcome, specifically whether CR occurred within 90 days after the transplant, and it ignores when disease response occurred. 

Of all the clinical endpoints typically used in medical RCTs, OS is the least ambiguous and least subject to measurement biases. It is considered a highly reliable “gold standard” outcome, provided that no subsequent salvage therapies are given after disease progression and the measured OS event of death occurs either without measurable disease progression or shortly after disease progression. In this case, the statistical estimate for the relative treatment effect on the OS endpoint is physically justifiable by random allocation to treatment and is strongly associated with PFS time (Figure 12A). If, instead, salvage therapy is given at or shortly after the time of progression, then the effect of the frontline treatment on OS time is confounded by the effect of the salvage treatment selection on the time from progression to death. In this case, randomization between different frontline treatments cannot provide a fair comparison, because OS time may take one of two forms. It is either the time of death without progression, which depends on the frontline treatment assigned by randomization, or it is the sum of the time to progression and the subsequent time from progression to death, which depends on both the frontline and salvage treatment. (Figure 12B). Consequently, the distribution of OS time is the distribution of the sum of two event times, the first depending only on the frontline treatment and the second depending on the pair (frontline, salvage), where “salvage” refers not only to the second treatment given but also to the adaptive rule used to choose it based on patient characteristics at progression, including time to progression. This pair is an example of a dynamic treatment regime (DTR), and in this case, comparisons should be made between pairs of possible DTRs, rather than only frontline treatments [187,188,189,190,191,192,193].

Estimates for the relative treatment effect of the first intermediate clinical outcome, such as PFS or DFS, following the initial random allocation, are physically justifiable by randomization. Prognostic variables influencing these outcomes can be used as blocking variables to further increase the precision of the intermediate endpoint estimates. However, such standard RCT analysis models are insufficient for proper estimation of OS. As illustrated in Figure 12B,C, the decision of which subsequent therapies to offer is confounded by each patient’s covariates at that time point. This systematic confounding is similar to the confounding that occurs in observational studies [113]. It would therefore be misleading to analyze OS from such trials using approaches meant for RCTs with completely randomized treatment allocation. 

As an example, contemporary adjuvant therapy RCTs for ccRCC, such as KEYNOTE-564, can block and adjust for variables prognostic of recurrence risk, such as tumor stage and yield comparative estimates of DFS difference that are physically justifiable by the random allocation procedure (Figure 12C) [54]. However, this physical justification applies to the OS only for those scenarios where patients die without disease recurrence. For the majority of patients, who may die after experiencing disease recurrence, subsequent salvage therapies will be administered and the decision between such options will be influenced by confounders such as the IMDC score at the time of subsequent treatment choice as recommended by organizations such as the National Comprehensive Cancer Network (NCCN) [194]. Statistical modeling of OS therefore needs to account for the frontline therapy, subsequent therapies administered, and confounders such as the IMDC score that influence the choice of therapy. This will generate the apples-to-apples comparisons between treatment regimens that clinicians need to estimate the potential OS outcomes for patients seen in the clinic depending on the treatment regimen chosen. For example, the OS of a patient with stage 3 ccRCC who was not treated with adjuvant therapy and received subsequent therapy with cabozantinib upon IMDC poor-risk disease recurrence should not be compared with that of a patient with stage 3 ccRCC who was treated with adjuvant therapy and received subsequent salvage therapy with cabozantinib upon IMDC favorable-risk disease recurrence. Instead, the proper comparator is a patient with stage 3 ccRCC who was treated with adjuvant therapy and received subsequent salvage therapy with cabozantinib upon IMDC poor-risk disease recurrence (Figure 12C). Depending on the context, additional analyses may be performed to test the causal hypothesis that adjuvant treatment allocation may influence the IMDC risk at recurrence. These may be considered two-stage DTRs, denoted by (frontline therapy, salvage therapy), where randomization chooses the frontline therapy, but rules based on intermediate covariates and time to recurrence may be used to choose the salvage therapy [188,189,195]. In such settings, DTRs should be compared, not just frontline therapies, and proper OS estimation requires far more care and information on potential confounding effects than DFS. 

**Figure 13:**
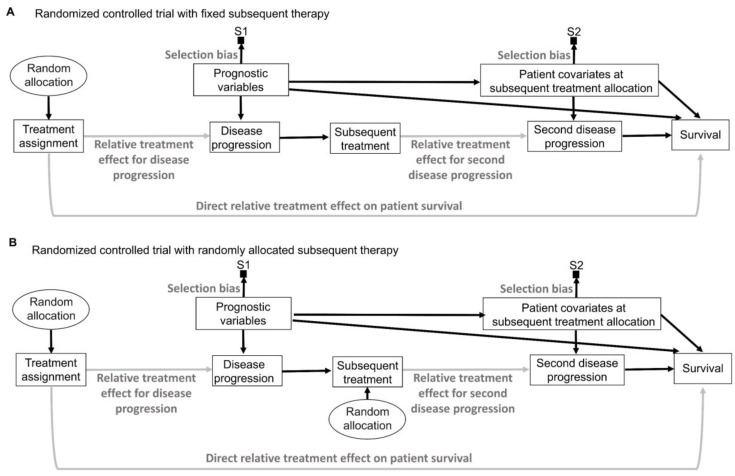
Selection diagrams representing the data-generating processes of clinical endpoints in RCTs to evaluate treatment regimes. (**A**) RCTs evaluating static treatment regimes prespecify a fixed subsequent treatment strategy that all enrolled patients will use upon disease progression to the randomly assigned first treatment. Thus, the only variable that influences whether a patient receives the subsequent treatment is the presence of disease progression to the first treatment. (**B**) RCTs evaluating dynamic treatment regimes may randomly allocate both the first and subsequent treatment assignment. This facilitates reliable estimation of the effect of sequential decision rules for the initial and subsequent therapy strategy to optimize long-term outcomes such as OS.

Contemporary therapeutic strategies are progressively shifting the management of diseases such as cancers toward more chronic rather than acute illnesses [196]. It is thus becoming more pertinent, both from a health policy and direct patient care perspective, to consider DTRs designed to improve OS, preserve quality of life, and minimize financial and other logistical costs. Powerful statistical models have been developed for this purpose [188,189,192,195,197,198,199,200,201,202]. However, to use these tools effectively, RCTs need to explicitly focus on minimizing confounding in their designs and collecting the necessary information to debias OS estimates. To remove the confounding effects on subsequent treatment choice, RCT designs may prespecify a fixed subsequent therapy regimen to be used, known as a “static treatment regime” (Figure 13A) [195]. Alternatively, random allocation can be performed both for the original treatment assignment and subsequent therapies in RCTs of DTRs, known as sequentially multiple randomized assignment trials (SMART) (Figure 13B) [191]. An early SMART specifically designed to evaluate well-defined DTRs was an RCT of advanced prostate cancer in which patients could be switched from a choice of four different initial combination chemotherapies to a second, different combination chemotherapy from the same set [197]. The design included re-randomization among the second-stage chemotherapies. Thus, rather than the conventional goal of simply comparing the four initial chemotherapies, the aim of the SMART design was to compare 12 different two-stage sequential decision rules aimed at maximizing long-term clinical benefit [197].

**Figure 14:**
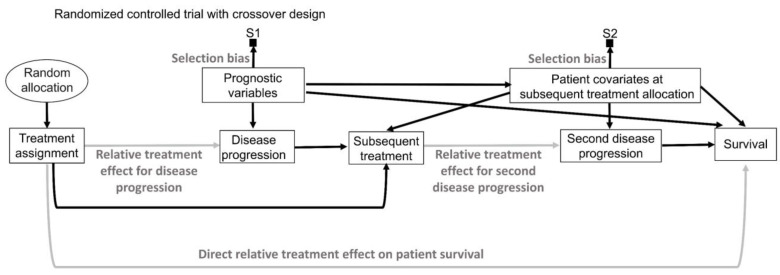
Selection diagram representing the data-generating processes of clinical endpoints in RCTs that allow crossover. Random allocation removes all other causal influences on the assignment of the first therapy, physically justifying the use of uncertainty estimates of the direct relative treatment effect on patient survival and the relative treatment effect for intermediate endpoints such as disease progression. These parameters are used for intermediate survival endpoints such as PFS or DFS. Due to potential crossover, the randomly assigned initial treatment will influence the choice of subsequent treatment. The effect of the original treatment assignment on survival will be mediated indirectly by such subsequent therapy choices and disease progression events, which can also be confounded by patient covariates at the time of subsequent treatment allocation. Depending on how the first treatment assignment influences the subsequent treatment during crossover, the OS parameter can be biased toward a false-positive or false-negative direction.

Crossover trials (Figure 14) are another example requiring careful consideration of the data-generating processes induced by the RCT design to ensure that OS estimates are not misleading [203]. In crossover trials, patients initially assigned to the control arm can be given investigational therapy after the first disease progression [204]. The justification may be ethical, to not deprive patients of potentially helpful therapy, or to prevent patient dropout from the RCT. However, whereas intermediate endpoints such as PFS may be unaffected by crossover, OS estimates may be falsely negative or positive depending on the RCT design [203,204]. An example of a false-positive OS signal occurred in the crossover RCT testing the platelet-derived growth factor receptor-α-blocking antibody olaratumab + doxorubicin versus doxorubicin alone in 133 patients with advanced soft tissue sarcoma [205]. The EMA and FDA granted approval of olaratumab in 2016, under the condition that additional RCT data would be provided in the future, based on the observed OS benefit in favor of the olaratumab arm compared with doxorubicin alone, despite a weak PFS signal. More specifically, for the secondary endpoint of OS, the trial yielded an HR estimate of 0.46, 95% CI 0.30–0.71, and *p* = 0.0003, corresponding to 12 bits of refutational information against the null hypothesis of no difference. The PFS signal was much weaker, with an HR estimate of 0.67, 95% CI 0.44–1.02, and *p* = 0.0615, corresponding to 4 bits of refutational information against the null hypothesis [205]. The false-positive OS signal due to crossover was shown by the subsequent ANNOUNCE trial, which did not allow for crossover, and revealed a deleterious effect of olaratumab in patients with advanced soft tissue sarcoma, as evidenced by a shorter PFS estimate and lack of OS benefit [206]. These results prompted the market withdrawal of olaratumab. The key flaw with the OS estimation in the original RCT was that 46% of the subjects in the control arm crossed over after progression to receive olaratumab monotherapy, while patients in the experimental arm sought out potentially effective second-line regimens [205]. Of note, patients in the experimental arm did not receive olaratumab monotherapy but the combination of olaratumab with the established active drug doxorubicin. Conversely, olaratumab alone was offered as subsequent therapy to patients in the control arm. One can consider employing a different statistical model with the hope of generating valid inferences. Using a Bayesian model with the winner’s curse prior derived from the 23,551 medical RCTs included in the CDSR [29,30,31], the posterior probability that the olaratumab arm yielded worse OS than the control was only 0.23%, while the posterior probability that olaratumab yielded worse PFS was 8.4%. Therefore, a strong signal persisted for a longer OS under the olaratumab arm in both the frequentist and Bayesian analyses, showing that trial data with a faulty design often cannot be salvaged by statistical analyses.

From a practical perspective, we should always consider the trial design and underlying data-generating process of trials to guide our statistical analysis models. In RCTs where no subsequent therapies are available (Figure 12A), standard statistical analyses can be used to compare OS. However, more careful modeling is required to compute OS estimates from RCTs when subsequent therapies are available, whereas intermediate endpoints such as PFS and DFS can be estimated reliably using standard methodologies (Figure 12B,C). Ideally, the intermediate endpoints and OS should point in the same direction. While it is unusual for a clinically active therapy to yield a negative signal for intermediate clinical endpoints and a positive signal for OS, or vice versa, such discrepancies may occur in moderate-sized trials due to the play of chance and should prompt further investigation [19,203]. Furthermore, it is not uncommon for an active therapy to yield a positive signal for an intermediate endpoint and an inconclusive result, as opposed to a negative signal, for OS, particularly in more indolent illnesses that require rigorous long-term follow-up for the OS event [19,207]. Careful assessment of the data is needed if the intermediate endpoint yields a positive signal in favor of the investigational treatment but OS shows the opposite signal in favor of the control arm. 

## 21. Synergy, Additivity, and Independence

With the development of a diverse portfolio of new therapies, the challenge has arisen to determine whether and how we can combine such agents and/or sequentially administer them as components of DTRs to maximize long-term clinical benefit [208]. The rationale behind combination therapies in fields such as infectious diseases and oncology is that microbes and cancer cells may develop resistance to a single drug whereas combinations can fully eradicate such heterogeneous populations prior to developing resistance [209,210,211,212,213,214]. Two combined drugs are considered to be additive when the half-dose of each drug in combination is equally as effective as the full dose of one drug alone [209]. The combination effect is synergistic or antagonistic when it is respectively found to yield better or worse efficacy than would be expected assuming additivity [209,215]. Additivity, synergy, and antagonism may be measured preclinically using measures of drug potency, such as the half-maximal inhibitory concentration (IC_50_), and efficacy, such as fractional cancer cell kill, to generate dose-response curves. However, commonly obtained dose-response data using survival outcomes in RCTs are often insufficient to determine additivity, synergy, or antagonism [209]. This highlights the need for careful dose-finding of therapy combinations in the early phases of development [4,139,216,217,218] and elucidation of patient-specific differences in drug pharmacokinetics and pharmacodynamics [219,220]. Tailored RCT designs such as factorial RCTs can be used to efficiently determine the contribution of each therapy by randomly allocating participants to receive neither, one or the other, or both interventions [8,221]. 

Notably, the activity in the RCTs of most FDA-approved drug combinations in oncology can be sufficiently explained by the concept of independence in the absence of drug additivity or synergy [209,222,223]. The mechanism of independence was first postulated in the trials conducted by the Acute Leukemia Group B (ALGB) and stipulates that each patient treated with a combination therapy can respond to only one of the two drugs and not both [209,224]. The implication is that drug combinations give each patient more chances of being exposed to the one drug that will be effective for them [209,222,223]. Furthermore, the independence mechanism stipulates that potent monotherapy clinical activity should be observed for each agent prior to considering their combination and that each agent in the combination should be administered at the maximal tolerated dosing [209,222]. In scenarios where independence is the mechanism behind the observed activity of a drug combination, the sequential use of these drugs alone, compared with their simultaneous combination, should also be carefully assessed. If it is found that sequential therapy with each agent alone yields similar or improved long-term clinical outcomes compared with the combination, then the adoption of sequential strategies may minimize unnecessary toxicities from intensive combination therapies. In such scenarios, combination approaches should be used only if rapid responses are desirable due to aggressive disease presentation that precludes the use of sequential strategies. In addition, combination therapies should be avoided for drugs known to have strong cross-resistance leading to highly correlated responses. Combinations of agents with different mechanisms of action should instead be prioritized [222]. Notable exceptions whereby co-inhibition of one pathway yielded synergistic efficacy despite one agent being devoid of monotherapy activity include the combination of fluorouracil with leucovorin across diverse malignancies, as well as the combination of EGFR and BRAF inhibition for BRAF-mutated colorectal cancer [209].

Multiple statistical analyses over the past decades suggest that independence of the agents in a combination therapy enables robust prediction of the outcomes of most RCTs in oncology that use combination therapies, including cytotoxic chemotherapy regimens or newer targeted therapies and immunotherapy agents [209,222,223]. However, one must be mindful of the patient relevance-robustness tradeoff described above [68,138]. The independence assumption for combination therapies is likely to yield robust inferences in the patient populations treated in RCTs. However, the independence assumption may not be relevant to the individual patient encountered in the clinic, as some patients may benefit from drug combinations because each agent may eradicate different tumor cells within the heterogeneous cancer population and prevent the development of resistance. For this reason, patient-centered translational research should be incorporated into RCTs to identify biomarkers and mechanisms predictive of therapeutic response and resistance to each agent, even if independence can robustly predict outcomes at the population level [68,225]. This phenomenon also illustrates why independence cannot explain curative regimens established for germ cell tumors, leukemia, and lymphomas, which are more reliably modeled assuming additivity [209]. As discussed in the above section on endpoints, the endpoint of cure is distinct from survival endpoints such as OS time, as patients who are not cured can live chronically with their disease and die of other causes. Cure rates may be considered additional endpoints of RCTs testing combination regimens, with the limitation of the OS time required to declare a patient “cured” [226,227].

## 22. Systematic and Random Biases

When designing and interpreting RCTs, causal diagrams such as the selection diagrams we have used here are helpful in identifying the potential presence of systematic biases, also known as systematic errors [68]. These may include selection biases (Figure 6A,B), systematic confounding (Figure 6A,C), nonadherence to the assigned intervention (Figure 9), and crossover bias (Figure 14). There are many other sources of systematic errors that can compromise the internal validity of RCTs. Causal diagrams can also be used to illustrate the data-generating processes subject to such biases. Examples of these biases include mediator–outcome confounding [82]; performance bias, which can be addressed by blinding the participants and trialists to the assigned intervention [143,228]; detection bias due to systematic differences between groups in how outcomes are measured [143]; attrition bias due to systematic differences between groups in cases of study withdrawal leading to bias from informative censoring [143]; and immortal time bias, also known as guarantee time bias or survivor bias, which occurs when RCT participants cannot experience the outcome during a period of follow-up time such as when outcomes are compared between responders and nonresponders to the randomly allocated intervention [229,230].

On the other hand, causal diagrams fail to indicate certain systematic RCT biases, such as reporting biases due to differences between reported and unreported findings [143], as well as not choosing an appropriate concurrent control arm for the study [8,231]. Furthermore, there are many experimental design scenarios where additional work is needed to synthesize contemporary causal inference techniques, such as causal diagrams. This includes the RCT analysis approaches developed at the Rothamsted Research Station by Ronald Fisher, Frank Yates, and John Nelder to properly estimate uncertainty measures based on how the treatment and block structures are defined in the RCT [232,233]. 

In addition to systematic errors, random biases also can occur in RCTs [234]. As shown in Figure 6A,B, random treatment allocation can remove systematic confounding, also known as confounding “in expectation” [235], influencing treatment assignment and outcomes. However, random confounding, also known as “realized” confounding [235], can still occur and is defined as the difference between the observed relative treatment effect in the actual RCT and the expected relative treatment effect on average over repetitions of this RCT [71,236]. As described earlier, such random confounding may occur because the random treatment allocation in an RCT generates observed imbalances in prognostic variables between the treatment groups [234]. Uncertainty estimates of relative treatment effects in RCTs are designed to account for such random errors induced by the randomization procedure [101]. Additional modifications of causal diagrams, such as single-world intervention graphs (SWIGs), have been proposed to more reliably investigate the effect of such random errors in forward and reverse causal inferences by explicitly representing factual, counterfactual, and potential outcome considerations [237,238,239]. 

## 23. Conclusions

The present comprehensive overview has emphasized that the defining feature of RCTs is random allocation, which justifies the estimation of a comparative treatment effect using measures of uncertainty such as *p*-values and CIs, and not random sampling, which would justify group-specific measures of uncertainty. By focusing on this distinction, we have elucidated a number of concepts necessary for the proper interpretation of RCTs to inform patient care. 

## Figures and Tables

**Table 1:** Results typically presented in medical RCTs.

RCT Measure	Examples	Role in RCT Interpretation	Additional Comments
Uncertainty estimates for the outcome differences between groups	CIs for HR, RR, OR, mean survival difference, or 1-year risk reduction	The major goal of RCTs is to generate valid uncertainty estimates for the differences between groups (comparative inference). This is achieved via random allocation.	Point estimates can be extrapolated from uncertainty intervals
Point estimates for the outcome differences between groups	HR, OR, RR, mean survival difference, or 1-year risk reduction	The differences between groups are the focus of RCTs	Point estimates alone without uncertainty estimates can be misleading
*p* values for the outcome differences between groups	*p* value for the null hypothesis of HR = 1.0	Refutational signals for tested hypotheses (usually the null hypothesis) and the background assumptions of the embedded statistical models	Can be converted into bits of refutational information (S values)
Group-specific measures	Median or mean survival, objective response rate, 1-year survival probability for each group	Descriptive measures providing information on the characteristics of the enrolled patients	Uncertainty measures such as SEs and CIs are valid in RCTs where random sampling has also been performed. Otherwise, measures of variability such as standard deviation or interquartile range are more appropriate.

CIs, confidence intervals; HRs, hazard ratios; ORs, odds ratios; RCT, randomized controlled trial; RRs, risk ratios; SEs, standard errors.

**Table 2:** Differences between random sampling and random allocation.

Goal	Approach Used in Random Sampling	Approach Used in Random Allocation	Additional Comments
Study design	Sampling theory	Experimental design	Random allocation may refer to random treatment assignment in RCTs, natural genetic variation in Mendelian randomization, or other natural random allocation processes used as instrumental variables
Describe the population of enrolled patients	Sample	Cohort	Cohorts of patients are not randomly sampled. They are randomly allocated to different exposures such as a treatment or control.
Use of uncertainty measures	Justified for group-specific parameters	Justified for comparative parameters representing differences between groups	Measures of variability such as interquartile range and standard deviation are preferred for group-specific parameters in the absence of random sampling
External validity	Generalizability from sample to broader population	Transportability from cohort to target population	Refers to the extension of knowledge between one population (sample or cohort) to another
Study underserved populations or minorities	Representative sampling	Representative causal mechanisms	Ethical oversight is warranted to ensure inclusiveness of RCTs with the goal to reduce healthcare disparities
Mitigate imbalances induced by the random procedure	Stratification	Blocking	Covariate adjustment can also account for random imbalances in RCTs

RCT, randomized controlled trial.

## Supplementary Materials

The following supporting information can be downloaded at: https://www.mdpi.com/article/10.3390/cancers15194674/s1, Supplementary File S1: Converting *p* values and CIs into bits. Supplementary File S2: Forest plot indifference zone estimation.
